# Pericytes Enrich the Basement Membrane and Reduce Neutrophil Transmigration in an In Vitro Model of Peripheral Inflammation at the Blood–Brain Barrier

**DOI:** 10.34133/bmr.0081

**Published:** 2024-10-03

**Authors:** Molly C. McCloskey, S. Danial Ahmad, Louis P. Widom, Pelin Kasap, Benjamin D. Gastfriend, Eric V. Shusta, Sean P. Palecek, Britta Engelhardt, Thomas R. Gaborski, Jonathan Flax, Richard E. Waugh, James L. McGrath

**Affiliations:** ^1^ Department of Biomedical Engineering, University of Rochester, Rochester NY, USA.; ^2^ Department of Biomedical Engineering, Rochester Institute of Technology, Rochester NY, USA.; ^3^ Theodor Kocher Institute, University of Bern, Bern, Switzerland.; ^4^ Department of Chemical and Biological Engineering, University of Wisconsin–Madison, Madison, WI, USA.; ^5^ Departments of Pharmacology and Neurosciences, University of California, San Diego, La Jolla, CA, USA.; ^6^ Department of Neurological Surgery, University of Wisconsin–Madison, Madison, WI, USA.

## Abstract

Sepsis is the most lethal and expensive condition treated in intensive care units. Sepsis survivors frequently suffer long-term cognitive impairment, which has been linked to the breakdown of the blood–brain barrier (BBB) during a sepsis-associated “cytokine storm”. Because animal models poorly recapitulate sepsis pathophysiology, human models are needed to understand sepsis-associated brain injury and to develop novel therapeutic strategies. With the concurrent emergence of tissue chip technologies and the maturation of protocols for human induced pluripotent stem cell (hiPSC), we can now develop advanced in vitro models of the human BBB and immune system to understand the relationship between systemic inflammation and brain injury. Here, we present a BBB model of the primary barrier developed on the μSiM (microphysiological system enabled by an ultrathin silicon nanomembrane) tissue chip platform. The model features isogenically matched hiPSC-derived extended endothelial culture method brain microvascular endothelial cell-like cells (EECM-BMEC-like cells) and brain pericyte-like cells (BPLCs) in a back-to-back coculture separated by the ultrathin (100 nm) membrane. Both endothelial monocultures and cocultures with pericytes responded to sepsis-like stimuli, with increased small-molecule permeability, although no differences were detected between culture conditions. Conversely, BPLC coculture reduced the number of neutrophils that crossed the EECM-BMEC-like cell monolayer under sepsis-like stimulation. Interestingly, this barrier protection was not seen when the stimulus originated from the tissue side. Our studies are consistent with the reported role for pericytes in regulating leukocyte trafficking during sepsis but indicate that EECM-BMEC-like cells alone are sufficient to maintain the restrictive small-molecule permeability of the BBB.

## Introduction

Sepsis, defined as a “life threatening organ dysfunction caused by a dysregulated host response to infection”, [1] remains one of the intensive care unit’s most deadly and costly conditions [2]. Even though treatments have improved survival rate, the long-term effects remain a burden to many sepsis survivors. While these effects vary from patient to patient, approximately one-third of sepsis survivors experience cognitive deficits that can last over 1 year and are associated with increased mortality [3]. Unfortunately, there is a paucity of therapeutic interventions to address this burden [3]. Identifying viable drug targets has been challenging, and animal models of sepsis have had poor translational relevance [4,5]. The emergence of tissue chip systems and the development of human induced pluripotent stem cell (hiPSC) technology now provide opportunities for more physiologically relevant, human-based in vitro models of sepsis and its deleterious impacts on the functions of multiple organs. In the following studies, we leverage the unique ability of porous membrane-containing tissue chip platforms to assess the role of vascular support cells (i.e., pericytes) and directional cytokine stimulation in the response of the brain to sepsis-like inflammation compared to local infections. Utilizing ultrathin nanomembranes with glass-like imaging properties, we are able to track immune cell migration in real time. To our knowledge, this is the first study combining these questions into a single coculture platform.

During normal inflammation, factors such as cytokines and chemokines are released from affected tissue to promote resident immune cell activation and circulating immune cell infiltration. On the contrary, during sepsis and other types of systemic inflammation, a “cytokine storm”, along with bacterial or viral antigens, enter the bloodstream, causing endothelial dysfunction and aberrant immune cell migration into uninfected tissues, ultimately leading to organ damage and even death [6]. Although the brain is traditionally considered “immune-privileged”, neutrophils and monocytes cross the blood–brain barrier (BBB) during systemic inflammation and are thought to contribute to the cognitive decline seen in sepsis survivors [7,8]. Furthermore, individuals with disorders that involve the BBB (Alzheimer’s disease, Parkinson’s disease, and multiple sclerosis) are at greater risk for cognitive decline following sepsis [9].

While endothelial dysfunction during sepsis is well established, the role of pericytes, the mural cells of microvessels, is understudied. Microvessels within the brain have the highest ratio of pericytes to endothelial cells in the body, with 70% to 80% vessel coverage [10]. Pericytes have well-established roles in barrier stabilization, endothelial cell polarization, and response to inflammation [11]. Their influence comes via paracrine signaling, cell–cell communications, and communication through the basement membrane shared with endothelial cells, which the pericytes both help deposit and embed themselves within [12]. Studies have elucidated multiple inflammatory roles of pericytes, including guiding immune cell migration, directing microglial polarization, and phagocytosis. They respond to sepsis-like stimuli and secrete inflammatory mediators such as cytokines, chemokines, reactive oxygen species, nitric oxide, and matrix metalloproteinases (MMPs) [13,14]. Further, pericyte loss occurs in the brain during sepsis [11,15] and is a hallmark of many central nervous system (CNS) diseases [16]. Thus, in this study, we sought to elucidate the role of human brain pericytes during sepsis-like inflammation to better understand how the pericyte loss seen in neurodegenerative disease and aging might exacerbate cognitive dysfunction following sepsis by impacting the function of the BBB.

To conduct these studies, we advanced a tissue chip of the BBB on the well-established μSiM (microphysiological system enabled by an ultrathin silicon nanomembrane) tissue chip platform [17,18]. Building on a study using only brain microvascular endothelial cells (BMECs) in the μSiM [18], we now add isogenically matched brain pericytes. Our coculture model features hiPSC-derived extended endothelial culture method brain microvascular endothelial cell-like cells (EECM-BMEC-like cells) [19] and brain pericyte-like cells (BPLCs) [20], which are separated solely by an ultrathin (<100 nm), highly porous (~15% porous) silicon-nitride nanomembrane with optical properties akin to glass. We compare this model, termed μSiM-BPB (BMEC-Pericyte Barrier), to our previously established EECM-BMEC-like cell monoculture model of the BBB, in the μSiM [18]. Advantages of the μSiM platform include the ability to visualize the structure and response of the composite EECM-BMEC-like cell-BPLC barrier, including its interaction with immune cells, with high-quality live and fixed imaging, small-molecule communication between the blood and brain compartments that is unimpeded by the membrane barrier, and access to a growing suite of protocols for the functional assessment of barrier function [18,21,22]. Other platforms are limited to permeability measurements without access to live imaging (e.g., Transwells) or live cell imaging without access to permeability (e.g., glass slides). Additionally, the nanomembrane technology provides a small-molecule permeability that is unrivaled in a membrane-based tissue chip platform, ensuring that barrier function is determined only by the cells and their deposited matrices.

Our studies show that BPLCs contribute substantially to the composition of the basement membrane but that this does not change EECM-BMEC-like cell permeability to the small molecule lucifer yellow (LY). The addition of BPLCs did, however, significantly reduce neutrophil diapedesis under sepsis-like conditions compared to EECM-BMEC-like cell monoculture. We note that the reduced immune cell migration seen in the coculture condition was not recapitulated when stimulation originated from the tissue-side, suggesting that pericytes may regulate neutrophil entry into the CNS in response to peripherally originating inflammatory challenges, such as in sepsis, but may not have the same protective function in the case of chronic neuroinflammation.

## Materials and Methods

### Silicon-nitride nanomembranes

The nanomembranes used in these studies were manufactured by SiMPore, Inc. (West Henrietta, NY) and consisted of 2 variants, nanoporous silicon nitride (NPN, NPSN100-1L) and dual-scale (DS, NPSN100-MP-1L-3.0LP). Both membrane types are ≈100 nm thick and feature nanopores ≈60-nm diameter with a nanoscale porosity of ≈15%. DS membranes also contain photolithographically patterned 3-μm-sized pores at a low density, resulting in additional microscale porosity of 0.75%. Each lot is characterized for pore size and density by SiMPore, Inc. and verified via electron microscopy.

### m-μSiM assembly

The component dimensions and assembly instructions for the modular μSiM (m-μSiM) have been described in detail previously [18,21]. Briefly, the top well (Component 1) and bottom channel (Component 2) were manufactured at ALine Inc. (Signal Hill, CA). Membrane chips and components were irreversibly bonded using pressure-sensitive adhesive. All assembly was done in a sterile environment (i.e., biosafety cabinet), and devices were further sterilized by ultraviolet (UV) for 20 min before use.

### μSiM-MVM assembly

To perform neutrophil trafficking studies, a hand-crafted version of the μSiM termed the μSiM-MVM (microvasculature mimetic) was manufactured with top and bottom microfluidic channels. These devices were manufactured under sterile conditions by following a layer-by-layer assembly protocol that has been described in detail previously [17]. Briefly, 300-μm-thick sheets of silicone gasket (Custom NuSil 4930, Trelleborg Sealing Solutions, Trelleborg, Sweden) were precision cut via a craft cutter (Silhouette America, Oren, UT) to create both membrane chip-sealing and holding layers. Next, 130-μm-thick sheets of adhesive transfer tape (468MP, 3M, Maplewood, MN) were cut into 2 microfluidic channels (top and bottom channel). These components were assembled into μSiM devices by attaching all components in a predefined order [17] on top of a glass coverslip (2980-244, Corning Inc., Corning, NY), with 15 min of UV-ozone treatment and 2 h of thermal incubation at 70 °C to irreversibly bond all layers together. Lastly, a polydimethylsiloxane layer (SYLGARD 184, DOW Chemical, Midland, MI) with fluidic inlets and outlets (1-mm diameter) was added to the top to serve as a support and cover for the device. For both monoculture and coculture trafficking studies, the devices used were equivalent except for the membrane interface. Monoculture studies were performed with NPN membranes that only feature nanoscale pores, while coculture studies were performed on DS membranes that feature both nanoscale and microscale pores. All devices were sterilized in a biosafety cabinet with UV for 20 min prior to use.

### Cell culture protocols

Cell cultures were maintained in a 37 °C incubator with 5% CO_2_/95% air and saturating humidity. All μSiM cultures were maintained in covered Petri dishes with a sterile water reservoir to reduce media evaporation. m-μSiM device culture protocols are described in detail in a prior publication [21], but brief methods are provided below for both m-μSiM and μSiM-MVM.

IMR90-4 Maintenance: IMR90-4 hiPSCs (WiCell, Madison Wisconsin) were maintained according to the manufacturer’s protocol in mTeSR1 (STEMCELL Technologies; for EECM-BMEC-like cell differentiation) or essential 8 medium (Gibco; for BPLC differentiation). Cells were maintained for at least 2 weeks in culture prior to initiating differentiation.

EECM-BMEC-like Cell Differentiation: EECM-BMEC-like cell differentiation was performed as previously described [19,23]. Briefly, cells were differentiated into endothelial progenitor cells by seeding onto tissue culture 12-well plates at a density of 100,000 cells per well on day −3 (D−3). On D0, media was changed to LaSR basal medium (Advanced DMEM/F12 (Gibco) with GlutaMAX Supplement (Gibco) and L-ascorbic acid) with 8 μM CHIR99021 (Sigma-Aldrich) on D0 and D1. On D5, CD31^+^ cells were sorted via magnetic-activated cell sorting. During the extended endothelial cell culture, cells were maintained on collagen IV (100 μg/ml, Sigma-Aldrich, reconstituted in water)-coated tissue culture 6-well plates in hECSR, which is human endothelial serum-free medium (Gibco) with serum free B-27 Supplement (1×, Gibco) and human fibroblast growth factor 2 (20 ng/ml, R&D Systems). Media was exchanged every 2 to 3 d, and cells were selectively passaged until passage 3 and used in assays between passages 3 and 7.

BPLC Differentiation: BPLC differentiation was performed as previously described [20,24]. Briefly, cells were differentiated into neural crest stem cells by seeding onto tissue culture 6-well plates at a density of 8.75 × 10^5^ cells per well on D-1 and maintaining in essential 6 medium (E6)-CSFD (E6 with CHIR99021, SB431542 [Tocris], human fibroblast growth factor 2 [R&D Systems], dorsomorphin [Sigma-Aldrich], and heparin [Sigma-Aldrich]) from D0 to D15. On D15, p75-NGFR^+^ cells were sorted via magnetic-activated cell sorting. Cells were then plated onto uncoated plates and on D16, and media was switched to E6 with 10% fetal bovine serum (FBS). Media was exchanged daily, and cells were used between D22 and D45 of differentiation.

EECM-BMEC-like Cell μSiM Monoculture: For EECM-BMEC-like cell monoculture in all μSiM devices, the top chambers of both m-μSiMs and μSiM-MVMs were first coated with a mixture of collagen type IV (400 μg/ml, Sigma-Aldrich, reconstituted in 0.5 mg/ml acetic acid) and bovine fibronectin (100 μg/ml, Gibco). Prior to cell seeding, the top chambers of both devices were washed with hECSR (100 μl for m-μSiM, 20 μl for μSiM-MVM), and the bottom chambers were also filled with the same media (20 μl for m-μSiM, 5 μl for μSiM-MVM). Two hours following seeding, media was replaced in the top chamber to remove nonadhered cells and subsequently replaced daily. All assays were performed on D5 to D8 of culture.

EECM-BMEC-like Cell and BPLC μSiM Coculture: For EECM-BMEC-like cell and BPLC coculture, μSiM devices were coated with 2 different coating solutions in each chamber. Top chambers were coated with a mixture of collagen type IV (400 μg/ml, Sigma-Aldrich, reconstituted in 0.5 mg/ml acetic acid) and bovine fibronectin (100 μg/ml, Gibco), using 100 μl for m-μSiMs and 5 μl for μSiM-MVMs. Bottom chambers were coated with 800 μg/ml collagen type IV (20 μl for m-μSiM and 5 μl for μSiM-MVM). The coating solutions were then washed with E6 + 10% FBS. For m-μSiMs, prior to BPLC seeding, the top well was filled with 50 μl of E6 + 10% FBS in order to prevent media spillage upon device inversion. Devices were held in hose clamps (VWR Cat. #MFLX06832-10 or Cole-Parmer Cat. #T-06832-10). BPLCs were seeded into the bottom chamber of both devices at a density of 14,000 cells/cm^2^ (resulting in a ~1:3 pericyte:BMEC ratio), and devices were flipped to allow cell adhesion onto the underside of the membrane surface. After 2 h, devices were flipped back over, and media in all chambers were replaced with E6 + 10% FBS. For m-μSiMs, the well volume was brought back up to 100 μl. The following day, media was replaced in both chambers of all devices with hECSR, and EECM-BMEC-like cells were seeded into the top chamber at 40,000 cells/cm^2^. After 2 h, media was replaced again to remove nonadhered cells, and hECSR was replaced daily. Assays were performed on D5 to D6 of endothelial cell culture.

Cytomix Stimulation: For sepsis-like stimulation, 16 to 20 h prior to assays, media in the top compartment was replaced with cytomix consisting of equimolar recombinant human tumor necrosis factor-α (TNF-α) (R&D Systems, 210TA), recombinant human interferon-γ (IFN-γ) (R&D Systems, 285IF), and recombinant human interleukin-1β (IL-1β) (R&D Systems, 201-LB). To model tissue-side inflammation, cytomix was added into the bottom channel of μSiM-MVM devices.

### Small-molecule permeability assay

Prior to conducting the permeability assay, confluency of the EECM-BMEC-like cells were examined under a phase microscope for gaps, and images were acquired for documentation using a 10× lens. The sampling-based assay has been described in detail prior [18,21]. Briefly, media in the top well was replaced with 100 μl of 150 μg/ml LY, 457 Da (Invitrogen), and μSiMs were incubated at 37 °C, 5% CO_2_ for 1 h. Following incubation, media from the well was removed, and a reservoir pipet tip containing 50 μl of media was added to one port. Media was then added back into the well to prevent disruption of the EECM-BMEC-like cell monolayer during sampling. A 50-μl volume from the bottom chamber was collected by reverse pipetting from the opposite port, added to a black 96-well plate, and fluorescence intensity was measured on a plate reader (TECAN, Männedorf, Switzerland). System permeability, ***P***_***s***_, was calculated using Eq. 1:

$$P_{S}=\frac{C_{t}*V}{C_{i}*t*A}$$ (1)

where ***C***_***t***_ is the concentration of fluorescent small molecule in the bottom channel at time ***t***, ***V*** is the volume transferred to the 96 well plate, ***C***_***i***_ is the initial concentration of fluorescent small molecule added to the top well, and ***A*** is membrane area (measured using Fiji). Endothelial permeability, ***P***_***e***_, was then calculated using Eq. 2:

$$\frac{1}{P_{e}}=\frac{1}{P_{S}}-\frac{1}{P_{M}}$$ (2)

where ***P***_***e***_ is the permeability coefficient relating to the endothelial monolayer, ***P***_***S***_ is the system permeability as calculated in Eq. 1, and ***P***_***M***_ is the system “membrane” permeability calculated on coated cell-free devices. For assays on DS membranes and NPN devices used for comparison to DS, system permeability is reported rather than endothelial permeability, since the properties of the DS membranes prevent collection of dye from the channel on coated control devices.

### Immunofluorescence staining

A complete list of antibodies can be found in Table S1, Supplementary Materials.

ICAM-1: For intercellular adhesion molecule-1 (ICAM-1) live cell staining, 100 μl of mouse anti-human ICAM-1 primary antibody diluted in hECSR was added to the top chamber of devices, and EECM-BMEC-like cells were incubated for 15 min at 37 °C, 5% CO_2_. Cells were washed with phosphate-buffered saline (PBS) in the top well, and then both chambers were fixed with 4% paraformaldehyde for 10 min at room temperature (RT). After washing both chambers 3 times with PBS, the top well was blocked for 20 min at RT with 5% goat serum (Invitrogen, Waltham, MA) containing 0.1% Triton X-100. Devices were then stained with secondary antibody goat anti-mouse immunoglobulin G (IgG) Alexa Fluor 488 diluted in blocking solution for 1 h at RT, and nuclei were stained with Hoechst 33342.

Images were acquired using a 10× lens (NA 0.30) on a Nikon Ti2 Eclipse inverted microscope (Nikon Corporation, Tokyo, Japan) and Zyla scientific complementary metal–oxide–semiconductor (sCMOS) (Andor Technology, Belfast, UK) camera or using a 10× lens (NA 0.45) on an Andor Spinning Disk Confocal microscope stage (Abingdon, UK) attached to a Nikon TiE microscope and a SONA sCMOS (Andor Technology, Belfast, UK) camera using wide-field mode. Images were processed using FIJI (ImageJ) software, and nonstimulated and stimulated images were linearly adjusted for equivalent intensity. For ICAM-1 fluorescence intensity quantification, mean fluorescence intensity was measured over the membrane region using FIJI software. Ratios were calculated by dividing mean fluorescence intensity of each image by the average mean fluorescence intensity of the nonstimulated devices for the respective experiment.

Claudin-5 and PDGFRβ: For Claudin-5 and platelet-derived growth factor receptor β (PDGFRβ) staining, EECM-BMEC-like cells cocultured with BPLCs were fixed with precooled (−20 °C) methanol in both the top well and bottom channel for 20 s and washed 3 times with PBS. Both chambers were then blocked for 20 min at RT with 5% goat serum containing 0.4% Triton X-100. Cells were stained with mouse anti-human claudin-5 and rabbit anti-human PDGFRβ primary antibodies diluted in blocking solution for 1 h at RT. Cells were washed 3 times with PBS and then stained with secondary antibodies, goat anti-mouse IgG Alexa Fluor 488, and goat anti-rabbit IgG Alexa Fluor 568 diluted in blocking solution for 1 h at RT. Nuclei were stained with Hoechst 33342.

Basement Membrane: For basement membrane live cell staining, primary antibodies, mouse anti-human collagen type IV Alexa Fluor 647, rabbit anti-human fibronectin Alexa Fluor 488, and rabbit anti-human laminin were diluted in hECSR with 10% FBS, added to both apical and basal chambers, and incubated for 2 h at 37 °C, 5% CO_2_. Both chambers were washed with PBS and fixed with 4% paraformaldehyde for 15 min at RT. After washing both chambers 3 times with PBS, the devices were blocked for 20 min at RT with 5% goat serum. Devices were then stained with secondary antibody goat anti-rabbit IgG Alexa Fluor 568 diluted in 5% goat serum for 1 h at RT, and nuclei were stained with Hoechst 33342.

Confocal images were acquired using a long working distance 40× lens (numerical aperture [NA] 0.55) on an Andor Spinning Disk Confocal microscope stage (Abingdon, UK) attached to a Nikon TiE microscope (Nikon Corporation, Tokyo, Japan) and a SONA sCMOS (Andor Technology, Belfast, UK) camera. Optical images were taken every 0.2 μm starting from below the pericyte layer to the top of the BMEC layer (5 μm above nanomembrane). For basement membrane imaging, a piezo stage was used, and the z-slice containing the nanomembrane was set as the z-axis origin (0 μm) to align different experimental z-stacks for analysis. The nanomembrane was located by focusing on the chip’s trench edge, which corresponded to the plane containing the nanomembrane. Confocal images were processed using Imaris software, and all basement membrane images were linearly adjusted for each channel for equivalent intensity. To plot basement membrane fluorescence intensity profiles, z-axis profiles for each channel were obtained in Fiji. The z location corresponding to the nanomembrane was set to 0 μm to align all data. For collagen type IV fluorescence intensity profiles, images were acquired on different devices than the rest of the data due to photobleaching of the antibody during imaging. The laser intensity and exposure time were reduced to minimize photobleaching for these devices.

### PMN isolation

The following protocol has been reviewed and approved by the University of Rochester Institutional Review Board. Primary human polymorphonuclear leukocytes (PMNs) were obtained via density gradient separation of whole blood that was drawn from healthy and consenting adult donors. Donors had blood drawn via veins in the arm (e.g., median cubital) that are commonly used for venipuncture procedures. Up to 10 ml of venous blood was deposited into sodium-heparin-coated vacuum tubes (B.D., Franklin Lakes, NJ). Blood samples were then cooled to RT (~20 min), and 3 ml of a density gradient solution (“1-Step Polymorphs”, Accurate Chemical & Scientific Co., Westbury, NY) were placed in a conical tube. After cooling, equivalent amounts of whole blood (3 ml) were gently layered on top of the density gradient solution, and the resulting combination was spun at 500xg for 30 min at 20 °C. Post centrifugation, only the PMN-rich layer was taken and deposited into a 15-ml conical tube, while the rest was discarded. Following isolation from the density gradient solution, the PMNs were washed twice by pelleting and resuspending (350xg for 10 min at 20 °C). Washing was performed with a buffer composed of sterile filtered Hank’s balanced salt solution (Ca^−^ and Mg^−^) mixed with 4-(2-hydroxyethyl) piperazine-1-ethanesulfonic acid (Hepes sodium salt) and lyophilized bovine serum albumin at 10 mM and 5 mg/ml, respectively. Red blood cells were eliminated from the PMN-rich solution via lysis utilizing a hypotonic gradient where 4.5 ml of one-sixth times PBS were added for 1 min, followed up by 1.5 ml of 4× PBS. Post red blood cell lysis, the pure PMN solution was washed once again, resuspended in 1 ml of wash buffer, and left on a rotating stand until the beginning of the experiment. Importantly, PMNs were used within 5 h of phlebotomy to minimize changes in cell behavior that occur gradually after phlebotomy.

### PMN trafficking videos

For both monoculture and coculture trafficking studies, μSiM-MVM devices were viewed using an inverted microscope (Nikon Ti2E, Nikon Corporation, Tokyo, Japan) coupled with an incubation stage set to a physiological temperature of 37 °C [22]. Briefly, 20 μl of PMN rich solution (3 million cells/ml) was introduced into the top channel of the device via pipette infusion. After PMNs settled, recording immediately began using a 40× long-working-distance (NA 0.55) lens in phase contrast. Cells located on the luminal surface of the monolayer appeared phase bright, while cells on the abluminal (tissue) side of the monolayer appeared phase dark. Images were taken once every 4 s (for an effective framerate of 0.25 Hz) for 30 min (450 frames total) using a high-resolution (2,048 × 2,048 pixels, 16-bit grayscale) sCMOS camera (Zyla 4.2, Andor Technology, Belfast, UK). After recording, videos were saved as image stacks (.TIF). Videos of monoculture studies were prepared for analysis with a semiautomated machine learning (ML) pipeline, while videos of coculture studies were analyzed manually. Both types of studies were analyzed using protocols described by Ahmad et al. [22].

### Computational resources and video processing

For monoculture PMN trafficking studies, the ML process was run on a 2017 iMac pro (Apple Inc., Cupertino, CA) with an 18-Core/36-thread (Xeon W, Intel Corporation, Santa Clara, CA) processor and 64 GB of RAM. Briefly, 16-bit grayscale .TIF stacks with a resolution of 2,048 × 2,048 pixels were compressed from ~3.5 to ~0.5 GB via bit reduction (to 8-bit) and bilinear interpolation (to a resolution of 1,024 × 1,024 pixels). After running the ML protocol, PMN parameters such as population counts, transmigration ratios, and tracking metrics (speed and persistence) were extracted. Cell speed is measured in micrometers per minute, while persistence time is measured in seconds and is used for describing trajectory directional stability [25].

Coculture PMN trafficking studies were analyzed manually for the same parameters with the same computer by using plugins commonly available within the FIJI software suite (Cell Counter and Manual Tracking). For manual cell counting, total PMN and transmigrated counts were tabulated on the first frame, and then on every subsequent 30th frame (i.e., frame 30, 60, 90 … 450). To make results comparable to monoculture studies on NPN membranes (no micropores), PMNs were marked as “transmigrated” if they went beyond the EECM-BMEC-like cell monolayer. For manual tracking, speed and persistence were determined by selecting 12 random PMNs in a video and tracking their centroids for as many frames as possible, with each track having at least 100 data points. A custom script written in Mathematica (Wolfram Research, Champaign, IL) was used to provide metrics that were comparable to the ML process output.

For tracking parameter extraction, mean square displacements, ***<d***^***2***^***>***, were calculated from centroids coordinates extracted from PMN trajectories and fit to a model of a persistent random walk that is used in cell migration studies [26] (Eq. 3). Parameters extracted were speed, ***S***, and persistence, ***P***. Note that ***n*** is dimensionality (*n* = 2).

$$<d^{2}>=nS^{2}\left[\mathrm{Pt}-P^{2}\left(1-e^{-t/P}\right)\right]<d^{2}>=nS^{2}\left[\mathrm{Pt}-P^{2}\left(1-e^{-t/P}\right)\right]$$ (3)

Note that persistence measures the ability of a cell to maintain direction [25,27,28], which is analogous to another frequently used measurement called “meandering index” [29]. Since persistence is derived from a model and is correlated with speed, we report this result instead.

### Statistical analysis

For all statistical analysis, GraphPad Prism software (GraphPad, La Jolla, CA) was used. For comparisons between groups in PMN trafficking studies, an ordinary 1-way analysis of variance (ANOVA) was used. For permeability assay comparisons with 1 independent variable and ICAM-1 fluorescence intensity comparisons, Brown–Forsythe and Welch ANOVA tests were used to account for heteroscedasticity of independent variables. To avoid errors attributed to “differences in nominal significance” [30], a 2-way ANOVA was used to make comparisons for data with 2 independent variables, followed by a Tukey post hoc test. To compare between stimulations for NPN and DS coculture studies, a 1-way ANOVA was used to compare column means. *P* ≤ 0.05 was considered statistically significant.

## Results

### EECM-BMEC-like cells cultured in μSiMs are responsive to sepsis-like stimuli

**Fig. 1.**
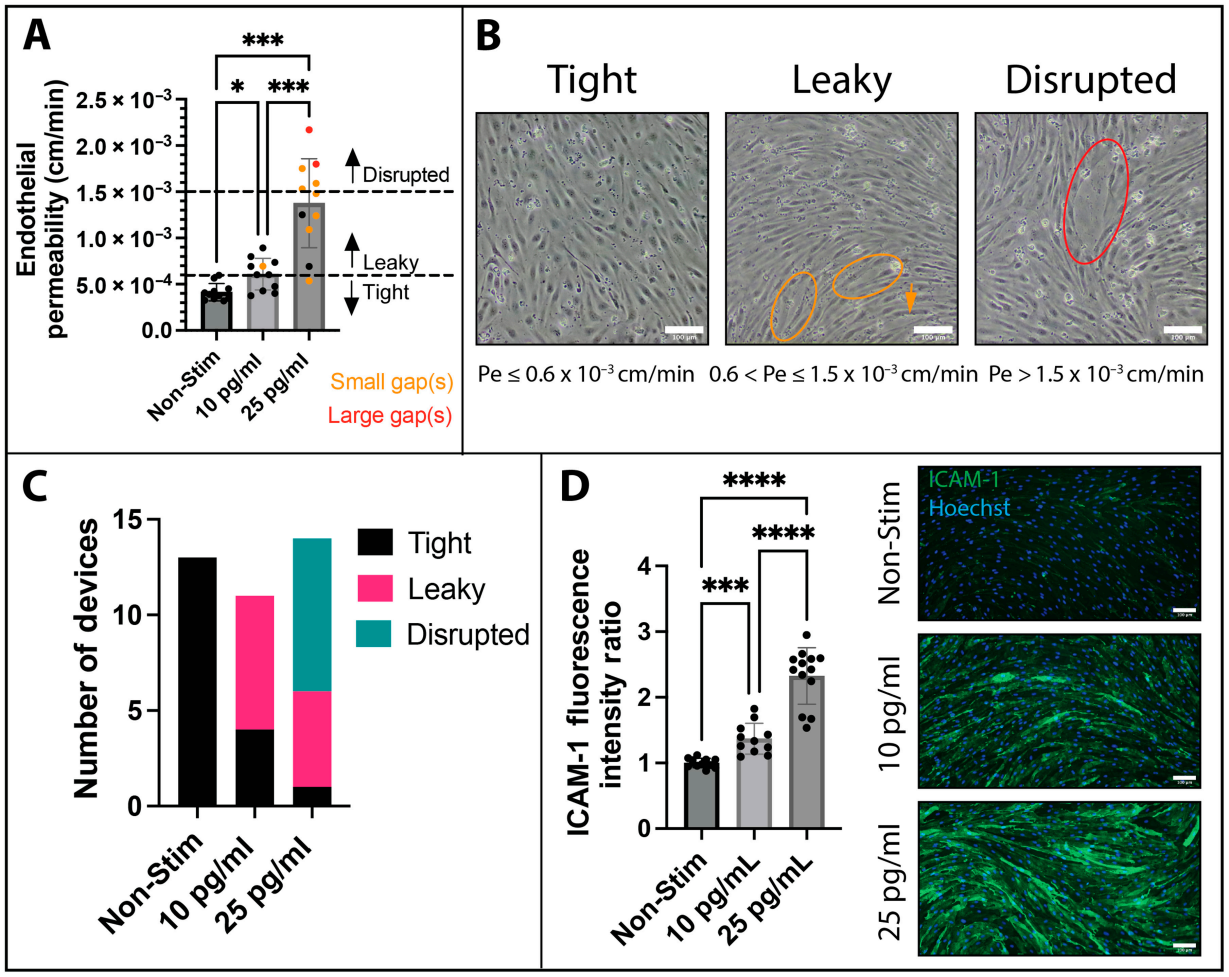
Characterization of EECM-BMEC-like cell monoculture responses to sepsis-like stimuli. EECM-BMEC-like cells were cultured in μSiMs for 6 d and either not stimulated (Non-Stim) or treated for 16 to 20 h with cytomix (equimolar TNF-α + IFN-𝛾 + IL-1β at 10 or 25 pg/ml each cytokine). (A) EECM-BMEC-like cell permeability was measured and plotted as mean ± SD. Orange data points indicate devices with small gap(s) (approximately the size of a single cell), and red data points indicate devices with large gap(s) (larger than the size of a single cell). *N* = 11 per group. Brown–Forsythe and Welch ANOVA tests were used. (B) Phase images of EECM-BMEC-like cells acquired prior to performing the permeability assay. Circled are small gaps (orange) and large gaps (red). Scale bar = 100 μm. (C) Endothelial permeability was categorized into “Tight” (Pe_LY_ ≤ 0.6 × 10^−3^ cm/min), “Leaky” (0.6 × 10^−3^ cm/min < Pe_LY_ ≤ 1.5 × 10^−3^ cm/min) and “Disrupted” (Pe_LY_ > 1.5 × 10^−3^ cm/min), and a contingency plot was made to highlight the differences in barrier categories between treatment groups. (D) Cells were stained for ICAM-1 (representative images, right), and mean fluorescence intensity over the membrane window was measured and normalized to nonstimulated devices from the respective experiment (plot, left). Scale bar = 100 μm. *N* = 11 to 13 per group. Brown–Forsythe and Welch ANOVA tests were used. Statistics: * = *P* ≤ 0.05, ** = *P* ≤ 0.01, *** = *P* ≤ 0.001, **** = *P* ≤ 0.0001, ns = not significant.

To mimic the cytokine storm that enters the brain through the peripheral circulation, we applied an established mixture known as “cytomix”, containing an equimolar mixture of TNF-α, IL-1β, and IFN-γ [31], to the apical compartment of μSiM devices. EECM-BMEC-like cells were stimulated for 16 to 20 h with 0, 10, and 25 pg/ml (per cytokine) of cytomix, which are within the ranges of measured serum cytokine levels in human septic patients [32,33] (Fig. 1). These values were selected after discovering that concentrations of 50 and 100 pg/ml cytomix were poorly tolerated, causing noticable cell loss (Fig. S1). We found a dose-dependent increase in small-molecule permeability to LY (457 Da) in response to cytomix (Fig. 1A), with an increase from Pe_LY_ = 0.41 ± 0.09 × 10^−3^ cm/min in nonstimulated devices to Pe_LY_ = 0.61 ± 0.17 × 10^−3^ cm/min at 10 pg/ml cytomix and to Pe_LY_ = 1.38 ± 0.48 × 10^−3^ cm/min at 25 pg/ml cytomix. Interestingly, with the imaging capabilities enabled by the μSiM, we were occasionally able to detect gap formation in the EECM-BMEC-like cell monolayer in response to cytokines (red and orange marked data points in Fig. 1A and B). Most gaps were small—less than the size of a single cell—and required careful inspection of the monolayer to find. The ability to see these gaps in a live BMEC monolayer with a cell height of 1 mm and below is a unique and important benefit of the high optical clarity offered by the μSiM. Transwells require fixation and staining to visualize the monolayer, and so similar gaps may be, or falsely assumed to be, artifacts of sample preparation. We examined the relationship between the identification of gaps and LY permeability and found the following. Beginning with a “tight” baseline in which no gaps are visible and Pe_LY_ ≤ 0.6 × 10^−3^ cm/min, the presence of a few small gaps across the membrane window corresponded to Pe_LY_ ≥ 0.6 × 10^−3^ cm/min but less than or equal to Pe_LY_ = 1.5 × 10^−3^ cm/min, a regime of permeability we will term “leaky”. The lower-end value is based on established measures of fully matured IMR90-4-derived EECM-BMEC-like cell monolayers [18,19]. Not all leaky devices displayed visible gaps. In some instances, weakening of the barrier in response to cytokine stimulation occurred without gap formation visible under inspection with a standard cell culture microscope using a 10× objective (NA 0.45). There were also instances of highly responsive monolayers, where large gaps (≥ cell-sized) or numerous small gaps were formed. As these tended to be associated with Pe_LY_ > 1.5 × 10^−3^ cm/min, we defined this as a third permeability regime for EECM-BMEC-like, which we term “disrupted”. In order to visualize the barrier properties of the EECM-BMEC monolayers in our 3 treatment groups, we created a contingency plot (Fig. 1C). Nonstimulated EECM-BMEC-like cell barriers were all found to be “tight”, with endothelial permeability for LY below the Pe_LY_ 0.6 × 10^−3^ cm/min threshold. Devices treated with 10 pg/ml cytomix were split between tight and leaky barriers, whereas most 25 pg/ml stimulated barriers were disrupted. Highly disrupted barriers exhibiting obvious cell loss were not sampled for permeability and are not included in Fig. 1A.

We also evaluated cytomix response of EECM-BMEC-like cell monolayers in terms of up-regulation of adhesion molecule ICAM-1 via immunostaining (Fig. 1D), which we have found to be a highly reliable marker of endothelial cell activation. Quantifying by microscopy as previously described [18], we found a significant dose-dependent increase in ICAM-1 fluorescence intensity across groups, with a ~1.4-fold increase in ICAM-1 fluorescence intensity in response to 10 pg/ml cytomix and ~2.3-fold increase in response to 25 pg/ml cytomix compared to nonstimulated controls as measured across the whole EECM-BMEC-like cell monolayer after live staining followed by fixation. The studies show for the first time that μSiM-cultured EECM-BMEC-like cells display dose-dependent functional responses to sepsis-like stimuli.

### Establishment of μSiM-BPB coculture model

**Fig. 2.**
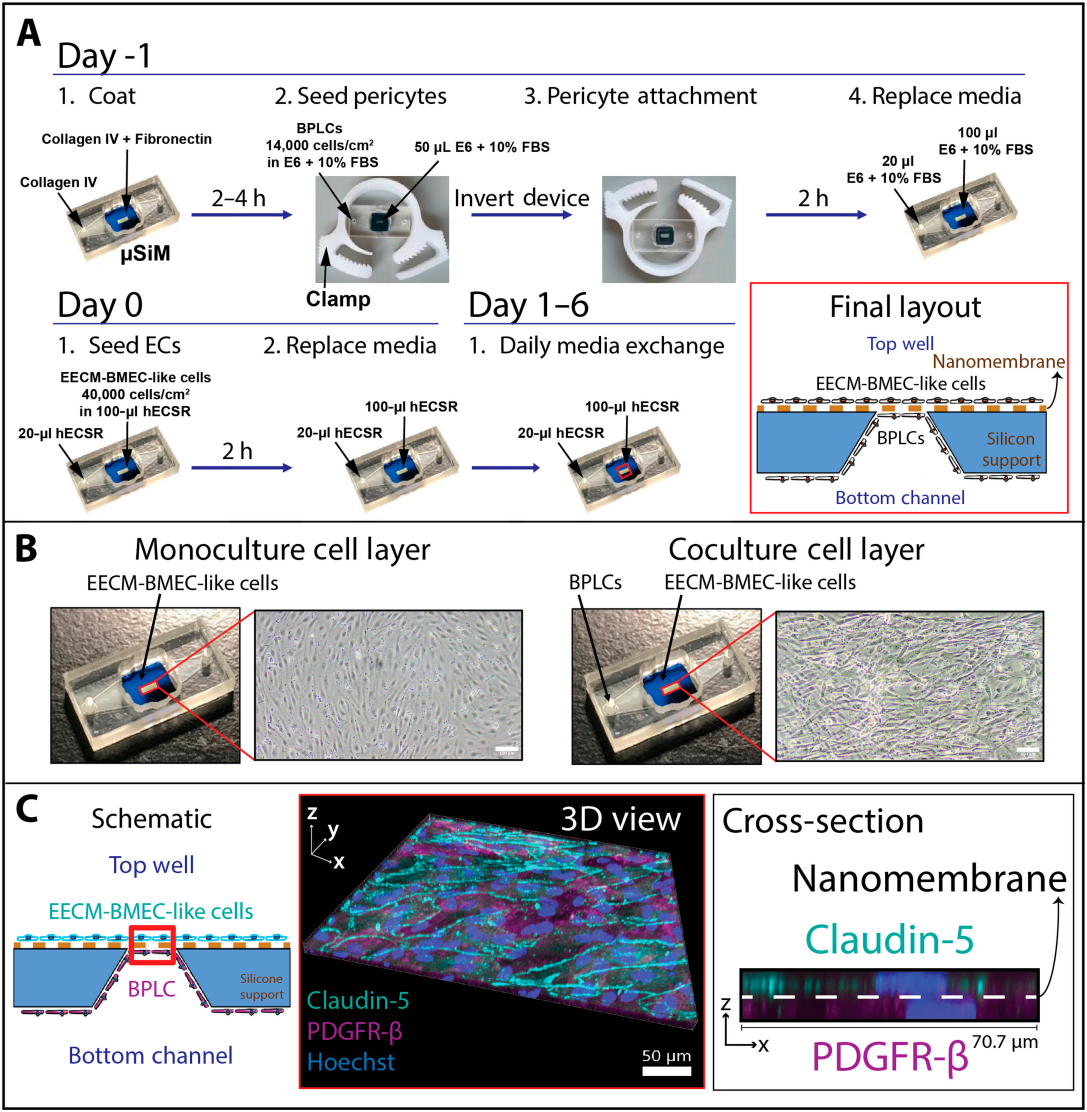
Establishment of μSiM coculture model, the μSiM-BPB. (A) Schematic of cell seeding protocol for EECM-BMEC-like cell and BPLC coculture in μSiMs. Red inset = side view of the final cell layout across the membrane region of the nanoporous chip. The blue region is the nontransparent silicon support, and the brown dash represents the nanoporous membrane (not drawn to scale). (B) Phase images of EECM-BMEC-like cell monolayers and coculture with BPLCs. Scale bar = 100 μm. (C) EECM-BMEC-like cells were cocultured with BPLCs and stained for brain endothelial cell tight junction marker Claudin-5 (teal), pericyte marker PDGFRβ (magenta), and nuclear stain Hoechst (blue). Confocal microscopy was used to acquire images across the nanoporous membrane region of the chip (red box). The 3D view (scale bar = 50 μm) and Cross-section (width = 70.7 μm) images demonstrate the close proximity of the 2 cell types, with the nanomembrane too thin to resolve in the stacks. Further, the Cross-section image shows the brain endothelial cell marker is restricted to the upper compartment and the pericyte marker is restricted to the bottom compartment.

To create a more physiological representation of the primary barrier between the blood and the brain, we sought to coculture BPLCs and EECM-BMEC-like cells on either side of the porous membrane in the μSiM (Fig. 2). BPLCs were validated by comparing RNA sequencing (RNAseq) and immunofluorescence staining for similarity to published BPLC expression profiles [20] and molecular expression of key pericyte markers (Fig. S2). As minimal differences existed between our in-house differentiated BPLCs and the previously published BPLCs and to primary pericytes, we considered the cells sufficient for our purposes.

We established the coculture by first seeding BPLCs into the bottom chamber of μSiM devices and flipping the devices to allow BPLCs to settle and adhere to the underside of the membranes (Fig. 2A). Devices were held in hose clamps (see Materials and Methods) so they could be more easily manipulated during this procedure. The following day, EECM-BMEC-like cells were seeded in the top chamber, and devices were maintained for an additional 6 d. In the final culture layout (red inset), the 2 cell types were separated only by the ~100-nm-thick nanoporous membrane at the porous region of the membrane chip (not drawn to scale). Both cell types could be visualized in the same focal plane and had distinct morphologies under standard phase-contrast microscopy. This important feature of the μSiM allowed us to monitor pericyte growth and density, with 50% to 100% coverage considered acceptable for subsequent assays (Fig. 2B). As BPLCs neared confluency, however, it became more difficult to monitor the endothelial cell layer and evaluate confluency of the EECM-BMEC-like cells in response to cytomix.

Confocal imaging then was used to evaluate the coculture model. Figure 2C illustrates the adjacent culture of the 2 cell types across the nanomembrane, with the brain endothelial tight junction marker, claudin-5, and pericyte marker, PDGFRβ, residing immediately on either side of an invisible plane occupied by the μSiM’s nanomembrane. Previous studies have demonstrated that the nanomembranes allow unhindered diffusion of small-molecule exchange, so long as the molecules are <50% the size of the nanopores (~60 nm) [34,35]. The cross-sectional image across a z-plane allows for further appreciation of the thinness of the nanomembrane, which is 2× thinner than the z-axis resolution of our confocal microscope.

### BPLCs contribute to basement membrane deposition

**Fig. 3.**
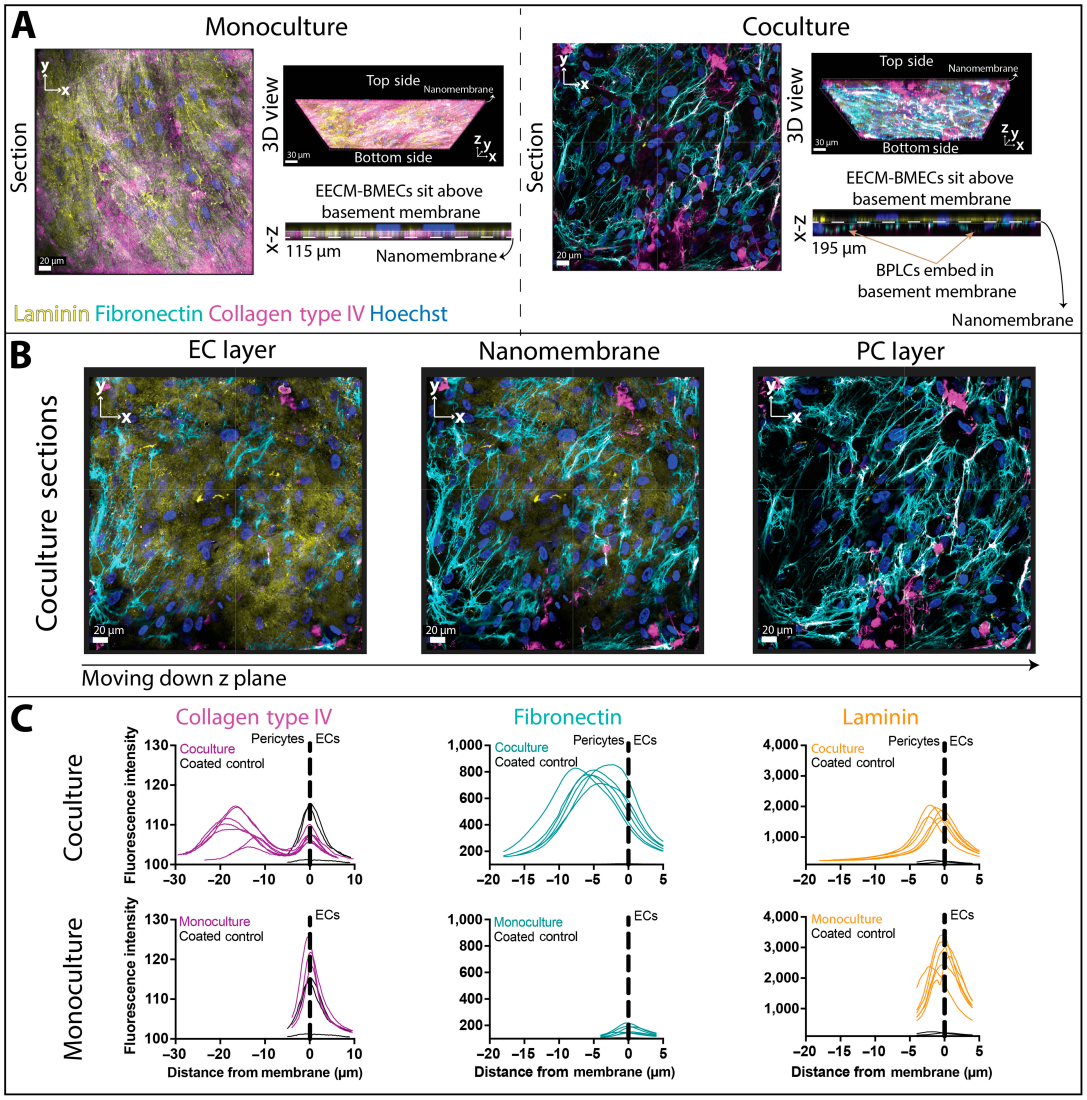
Evaluation of basement membrane deposition in the μSiM-BPB. EECM-BMEC-like cells (Monoculture) and EECM-BMEC-like cells and BPLCs (Coculture) were grown in μSiMs and stained for BM components collagen type IV (magenta), fibronectin (teal), and laminin (yellow), along with nuclear stain Hoechst (blue). Confocal microscopy was used to acquire images across the nanoporous membrane region. (A) Representative monoculture and coculture x-y “Section” (scale bar = 20 μm), “3D views” (scale bar = 30 μm), and “x-z” slices of confocal images. The 3D views show the entire confocal stack, focused below the nanomembrane, with the “Bottom side” indicating the pericyte layer and the “Top side” indicating the endothelial cell layer. The x-z slices are zoomed to show the location of the basement membrane components relative to each cell type. The approximate location of the nanomembrane is indicated. Endothelial cells sit above the BM deposited on the nanomembrane. Pericytes embed themselves within the BM. (B) Representative “Coculture sections” images moving down the z-axis. The leftmost image is focused along the z-axis within the top chamber (EC layer), the middle image is focused at the nanomembrane, and the rightmost image is focused within the bottom chamber (PC layer). Scale bar = 20 μm. (C) Mean fluorescence intensity across the z-axis was measured and plotted for each BM protein in coculture and endothelial cell monoculture, along with coated control devices (black). *N* = 2 to 3 devices for monoculture and coculture, and 1 to 2 devices for coated controls, with 1 to 3 images acquired per device.

One critical function of pericytes is deposition of key basement membrane proteins that provide the structural material of the microvascular wall [36]. To evaluate the basement membrane composition of the monoculture and coculture models, we stained for collagen type IV, fibronectin, and laminin (Fig. 3 and Fig. S3A). Figure 3A shows representative cross-sectional images of monoculture and coculture basement membrane composition, as well as a 3-dimensional (3D) view of the cultures and x-z slices. While all components were present in the EECM-BMEC-like cell monocultures, the basement membrane in the coculture BPLC layer was primarily composed of fibronectin and collagen type IV, with some colocalization of the 2 proteins. Furthermore, the coculture x-z slice illustrates that the BPLC position within the basement membrane is in accordance with its location in vivo. The EECM-BMEC-like cells were localized above their deposited matrix, whereas BPLCs embedded themselves within this shared basement membrane. While it is not possible to see the ultrathin nanomembrane in confocal imaging, it is important to note that, based on the location of EECM-BMEC-like cell nuclei in monocultures, the nanomembrane sits just below the EECM-BMEC-like cell-deposited basement membrane layer. The different compositions of EECM-BMEC-like cell- and BPLC-deposited proteins can be better appreciated from side-by-side images at different z-planes across the coculture device (Fig. 3B). While laminin was primarily visible in the EECM-BMEC-like cell layer and appeared to cover the membrane surface as a “patchy carpet”, fibronectin and collagen type IV were most prominent in the BPLC layer and were fibrous, with the transition appearing to occur at the nanomembrane.

To quantify the location of different matrix components in the BM, we extracted and plotted fluorescence intensity across z stacks for both monoculture and coculture images using FIJI (Fig. 3C). The z-axis origin was set to the image slice corresponding to the location of the nanomembrane during imaging (see Materials and Methods). This view makes it clear that there was collagen type IV in both cell layers, with fibronectin primarily located in the pericyte layer and laminin found almost exclusively adjacent to the endothelial cells. Small amounts of each protein, however, can be found in both cell layers. Interestingly, the monoculture-deposited fibronectin was markedly lower than the coculture values, even on the endothelial side of the membrane. Conversely, monoculture laminin was generally higher in monocultures than coculture.

Given that the membranes were precoated with collagen type IV and fibronectin prior to seeding, it was important to establish what contributions these coatings might make to the images. Coated control devices without cells were stained, imaged, and quantified. Although the collagen type IV fluorescence intensity peaks at the membrane in cell-seeded devices was similar to two of the coated controls, there was clearly a different structure to the collagen type IV layer in cell-seeded devices compared to the coated devices, in which most fluorescence seemed to come from antibody aggregates (Fig. S3B). Additionally, the dim staining seen in coated controls are clearly confined to the membrane and not extended into a 3D matrix as in the presence of cells. Thus, the initial membrane coatings do not compromise our ability to discern collagen IV deposition or remodeling by the cells. Similarly, fibronectin and laminin fluorescence intensities in cell-seeded devices were both well above measured ranges for the coated controls.

### BPLCs do not improve small-molecule permeability barrier properties of EECM-BMEC-like cells in the μSiM

**Fig. 4.**
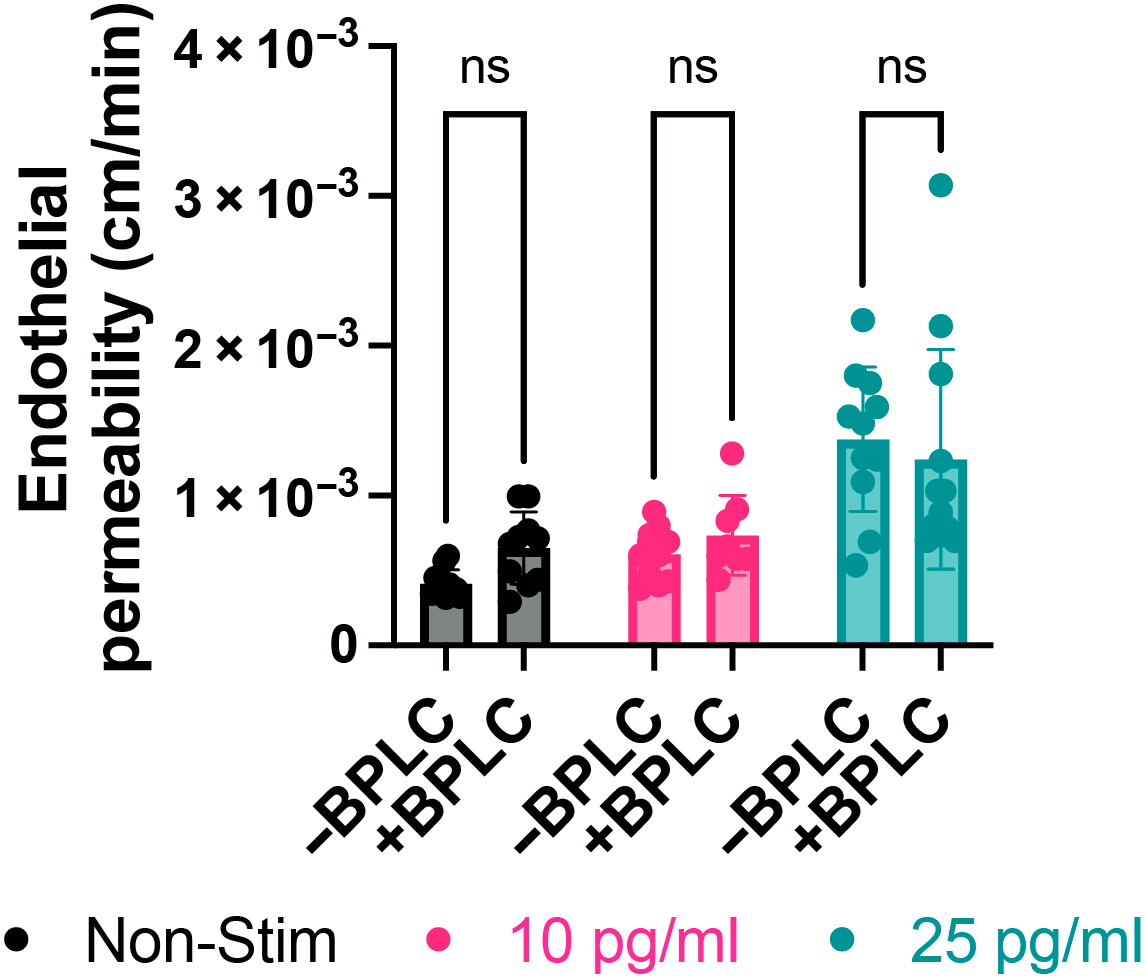
Evaluation of μSiM-BPB small-molecule permeability responses to sepsis-like stimuli. Endothelial permeability to LY was measured across EECM-BMEC-like cells without BPLCs (−BPLC) or cocultured with BPLCs (+BPLC) that were either not stimulated (Non-Stim, black) or treated for 16 to 20 h with cytomix at 10 pg/ml (pink) or 25 pg/ml (teal). Plotted are mean ± SD, *N* = 8 to 12 per group. A 2-way ANOVA was used with Tukey post hoc. Statistics: *P* ≤ 0.05 was considered significant, ns = not significant.

BPLCs have been shown to improve barrier properties of iPSC-derived BMEC-like cells and primary brain endothelial cells [20], and given their robust BM deposition, we measured small-molecule permeability (LY) in our coculture model under baseline (nonstimulated) conditions. We further evaluated if BPLCs might have a protective effect to the barrier breakdown in response to sepsis-like stimuli (Fig. 4). In accordance with our previous observations using a EECM-BMEC-like cell coculture with pericytes [19], coculture of EECM-BMEC-like cells with BPLCs did not result in lower permeability of nonstimulated endothelium. Our work additionally found that the dose-dependent changes in permeability in response to apical cytomix stimulation were comparable between coculture and monoculture.

### Apically stimulated EECM-BMEC-like cells are more permissive to PMN transmigration versus an equivalent basal stimulus

**Fig. 5.**
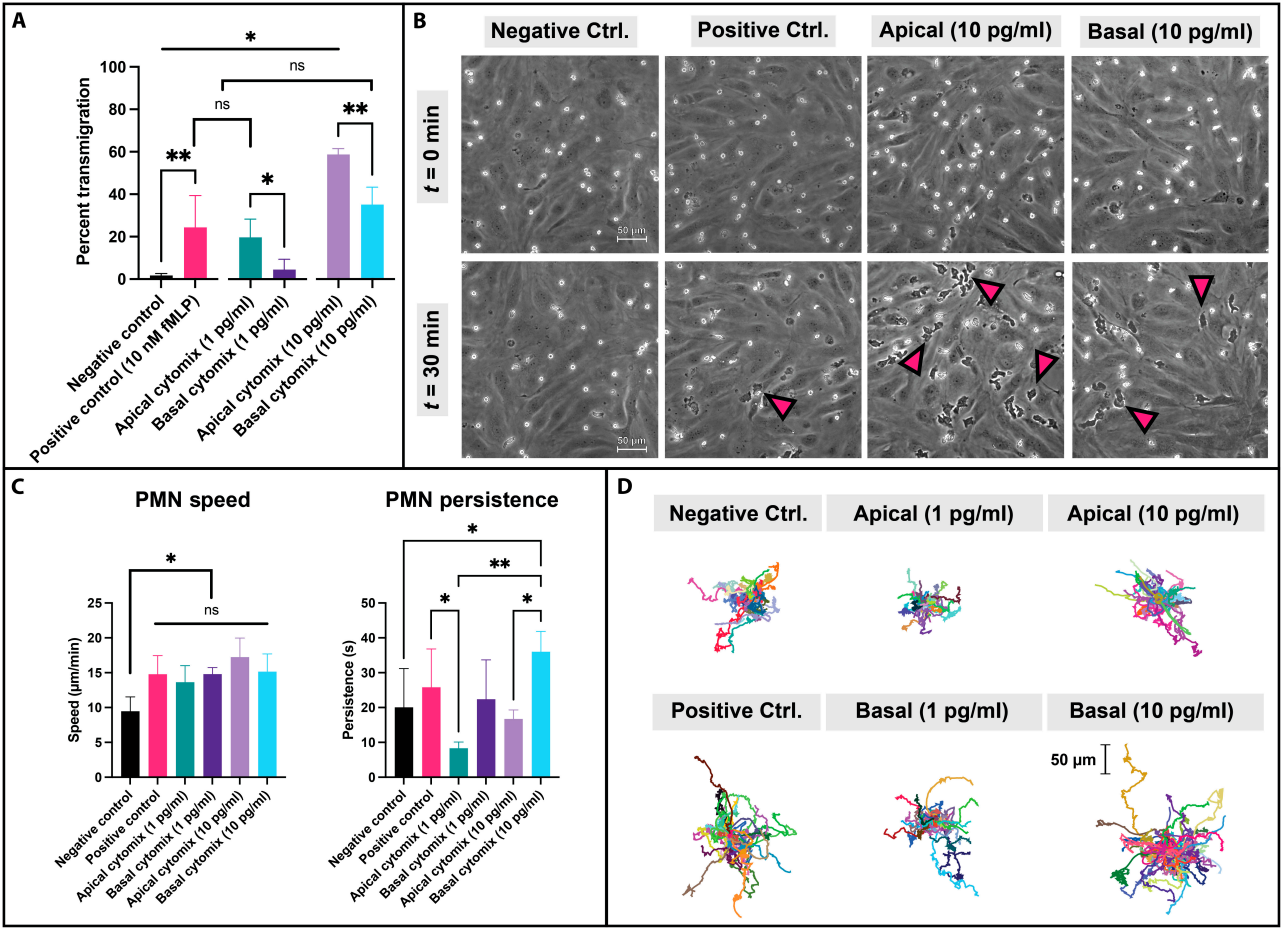
Apical cytomix stimulation of EECM-BMEC-like cell monolayers results in greater PMN transmigration. EECM-BMEC-like cells were cultured in μSiM-MVM devices for 6 d and exposed to cytomix (1 or 10 pg/ml) for 20 h prior to PMN introduction. Cytomix stimulation was performed in a sided manner to mimic sepsis-like or tissue-sided inflammation. (A) PMNs deposited upon apically stimulated endothelium engaged in significantly more transmigration compared to basally stimulated endothelium at both dosages. Notably, apically sided cytomix stimulation at 10 pg/ml facilitated a greater PMN transmigratory response than an fMLP gradient (~58% versus ~24% respectively). (B) Phase-contrast images (scale bar = 50 μm) of experimental conditions at *t* = 0 min and *t* = 30 min demonstrate robust differences in PMN response to cytomix. Large portions of the PMN population present in the apical cytomix (10 pg/ml) condition are transmigrated by 30 min, whereas PMNs in both basal and fMLP experiments appear to be primarily located on the luminal EECM-BMEC-like cell surface. Magenta arrows highlight transmigrated PMN examples. (C) Using an fMLP gradient or stimulating EECM-BMEC-like cells, regardless of dosage amount or direction, increases PMN crawling speed from a baseline of ~9 to ~15 μm/min. Persistence is found to be similar at low (1 pg/ml) cytomix levels regardless of stimulation direction, albeit with high variability between experimental replicates. Upon increasing cytomix to 10 pg/ml, PMNs crawling on apically stimulated endothelium remain significantly less persistent (~17 s) than those crawling on basally stimulated endothelium (~36 s). (D) Spider plots (scale bar = 50 μm) showing PMN trajectories with a universal origin help visualize motility differences across all experiments, where PMN activity depends on the direction and dosage of cytomix stimulation. All results are plotted as mean ± SD and analyzed with a 1-way ANOVA. Statistics: * = *P* ≤ 0.05, ** = *P* ≤ 0.01, ns = not significant.

To evaluate the transmigratory response of immune cells, we initially evaluated migration parameters across stimulated EECM-BMEC-like cell monolayers. Primary PMNs (i.e., neutrophils) were introduced into the top compartment of μSiM devices with EECM-BMEC-like cell monolayers. For these studies, the μSiM-MVM platform was used to minimize PMN settling time and image degradation from neutrophils settling into the open-well format of the μSiM (Fig. S4) [22,37]. Given the frequency of barrier disruption seen in response to a 25 pg/ml cytomix stimulation, 10 pg/ml was the maximum dose administered in all trafficking studies as leukocyte transmigration is partly driven by endothelial cell secreted endogenous chemokine gradients [17] that may be disrupted or equalized by the formation of large gaps in the EECM-BMEC-like cell monolayer. To investigate effects at a lower inflammation level, a group of devices also received a 1 pg/ml dose of cytomix (Fig. 5).

Following similar studies done previously on HUVEC monolayers [17], we added cytomix to either the apical or basal compartments to stimulate EECM-BMEC-like cells prior to the introduction of PMNs. While the apical stimulation mimics a sepsis-like challenge, the basal treatment can be thought of as a model of inflammatory signaling originating from perivascular tissue during neuroinflammation. Alongside these conditions, the bacterial peptide *N*-formylmethionyl-leucyl-phenylalanine (fMLP) was used as a potent PMN chemoattractant and added basally at 10 nM without any additional cytokines to serve as a positive control.

Steady-state transmigration ratios (established by ~15 min after the introduction of PMNs) for the negative control (no treatment) and positive control (basal fMLP) studies were 1.61% and 24.39% of total PMNs in the field of view (FOV), respectively. The cytomix studies demonstrated that PMN transmigration is both a dose- and spatially dependent phenomenon, as both higher doses and apically applied cytokine stimulation elicited greater PMN transmigration. Specifically, apically applied stimulation resulted in mean transmigration ratios of 19.68% at 1 pg/ml and 58.75% at 10 pg/ml, while basally applied stimulation elicited 4.51% at 1 pg/ml and 35.09% at 10 pg/ml (Fig. 5A). This can be seen in a phase-contrast imaging modality, where PMNs are shown to transmigrate significantly more under the apical cytomix stimulation condition at 10 pg/ml than any other experimental condition (Fig. 5B). Tracking parameters were then extracted from computationally generated PMN trajectories that were fit to a model of a random walk, where cell speed and persistence were compiled from a curve fit. Interestingly, PMNs demonstrated statistically similar speed under all experimental conditions and were slowest in the negative control (Fig. 5C). Persistence measurements displayed high variability, with PMNs demonstrating statistically similar persistence at 1 pg/ml stimulation regardless of stimulation direction and significantly higher persistence when EECM-BMEC-like cells were stimulated basally at 10 pg/ml (36.03 s) versus apically (16.74 s). PMN motility directly correlated with persistence, with spider plots of PMN trajectories in each stimulatory condition indicating that PMNs incorporated into basally stimulated devices (10 pg/ml) traveled more versus apically stimulated (10 pg/ml) ones (Fig. 5D). Taken together, these results demonstrate that PMNs more efficiently migrate across apically stimulated EECM-BMEC-like cell monolayers versus monolayers with a similar basal stimulus.

### DS membranes support a restrictive EECM-BMEC-like cell and BPLC coculture barrier

**Fig. 6.**
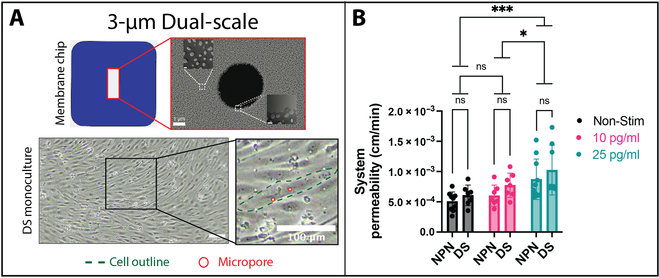
Establishment of the μSiM-BPB on 3-μm DS nanomembranes. (A) Scanning electron microscopy image of a membrane chip with a 3-μm DS nanomembrane (scale bar = 1 μm) and overlaid transmission electron microscopy images highlighting the structure of the nanoporous region (scale bar = 50 nm) and edge of the micropore (scale bar = 100 nm). Transmission electron microscopy image adapted from Salminen et al. [38] with permission; copyright WILEY. EECM-BMEC-like cells were cultured on the DS membrane and a phase image was acquired. The green dashed line is the outline of a single cell, and the red circles highlight micropores located underneath said cell. Scale bar = 100 μm. (B) EECM-BMEC-like cells were cocultured with BPLCs on nanoporous (NPN) or DS membranes and either not stimulated (Non-Stim, black) or treated for 16 to 20 h with cytomix at 10 pg/ml (pink) or 25 pg/ml (teal), and system permeability was measured. Plotted are mean ± SD, *N* = 7 to 10 per group. A 2-way ANOVA was used with Tukey post hoc comparing column means. Statistics: * = *P* ≤ 0.05, ** = *P* ≤ 0.01, *** = *P* ≤ 0.001, ns = not significant.

NPN membranes have many advantages for modeling barrier systems, but the nanoscale-sized pores prevent cell migration from the endothelial chamber to the pericyte chamber. Therefore, we used DS membranes into our coculture model to enable full leukocyte migration (Fig. 6). DS membranes maintain key advantages of NPN systems (high permeability and optical clarity) while also providing regularly spaced micropores for neutrophil extravasation into the pericyte chamber [18,38]. In these studies, we used 3-μm-diameter micropores at 0.75% micropore density. At this density, each endothelial cell was above 1 to 3 of the evenly spaced micropores (Fig. 6A). To confirm that the incorporation of micropores did not compromise barrier integrity, we conducted permeability experiments on NPN- and DS-grown cocultures and stimulated with cytomix (Fig. 6B). In this case, we are reporting system permeability rather than endothelial permeability because the high hydraulic conductivity of the micropores prevents collection of a coated control sample. There was no difference in nonstimulated permeability to LY detected between membrane types. Furthermore, cells showed a similar increase in permeability in response to cytomix. We also tested for differences in basement membrane deposition when using DS membranes. Cytomix-treated NPN and DS μSiM-BPBs were stained for collagen type IV, fibronectin, and laminin. There were no apparent differences in basement membrane deposition either between the 2 membrane types (Fig. S5, A and B), or after cytomix addition (Fig. S5, A and C).

Another advantage to using DS membranes in a coculture model is the possibility to establish physical connections between BMECs and pericytes. We observed the most dramatic evidence of the integration of these 2 layers when BPLCs were overgrown and became contractile during our optimization studies (Fig. S6). Overgrown BPLCs that were cultured on NPN contracted into clusters that were localized in the middle of the nanoporous membrane, while on DS surfaces, they remained strongly adhered to the membrane micropores when cocultured with EECM-BMEC-like cells. Importantly, this morphology was not seen when BPLCs were grown in monoculture on DS membranes, suggesting an interaction between the 2 cell types at the microporous interface.

### The addition of BPLCs eliminates the effect of sided endothelial cell stimulation on PMN migratory behavior

**Fig. 7.**
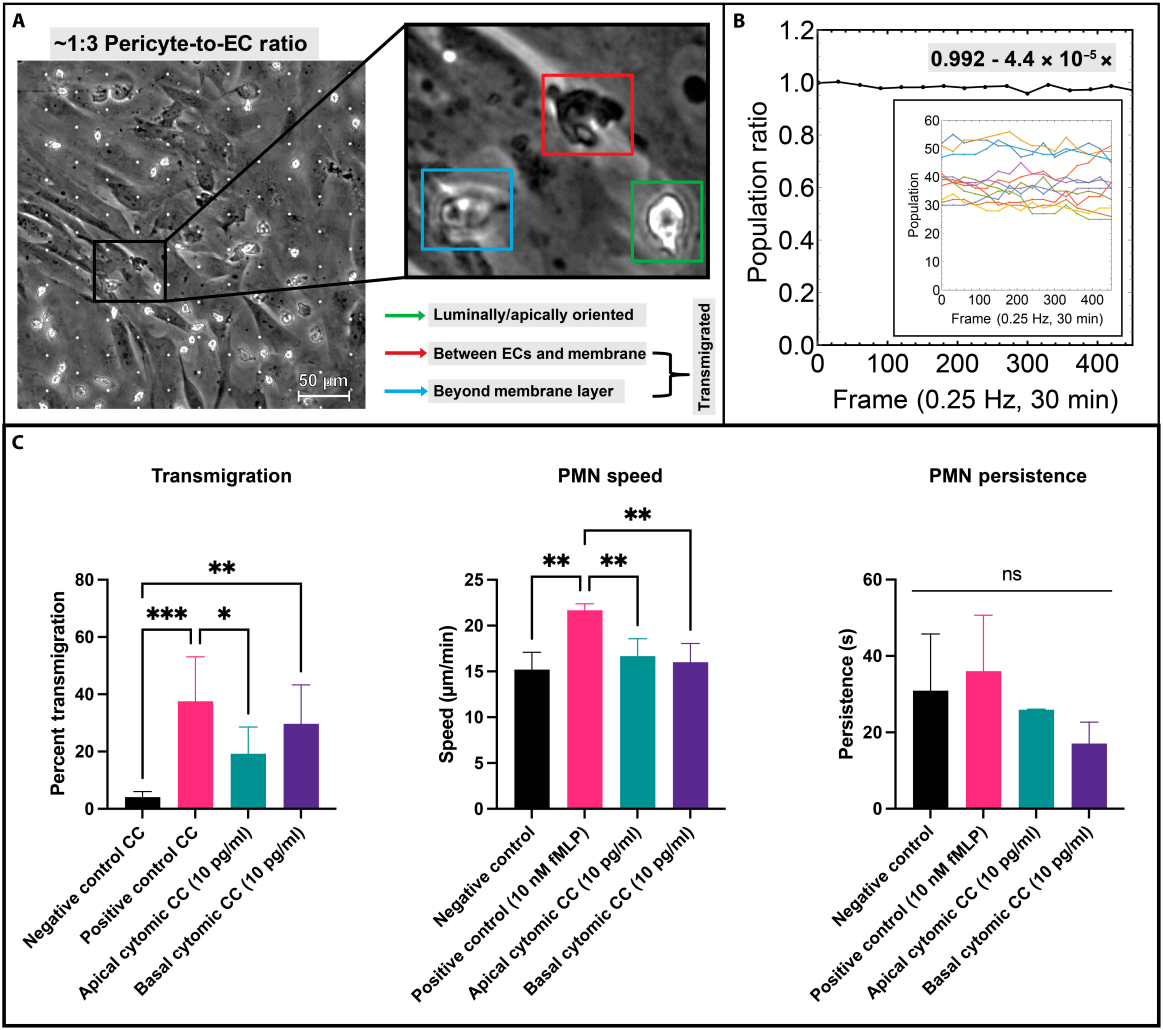
EECM-BMEC-like cell with BPLC coculture dampens PMN migration activity on apically stimulated devices. (A) The incorporation of DS membranes required retooling of the analytical protocol used for assessing PMN transmigratory behavior. Namely, manual analysis was used instead of computer vision protocols to account for the presence of both microscale pores and pericytes. To make coculture transmigration analysis comparable to monoculture transmigration studies, PMNs were considered “transmigrated” if they were spatially located below the EECM-BMEC-like cell layer (red and blue bounding boxes) and not luminally located (green bounding box). (B) The presence of DS micropores enables full PMN extravasation into the tissue compartment of μSiM devices. To ensure analytical compatibility with monoculture studies, where micropores were not present, population counts present in a recording FOV, across all experiments, were manually tabulated to see if population loss occurred over time (inset). When normalizing and averaging all results, we find that PMN population viewable in a FOV does not decline significantly over a 30-min period, thus making the transmigration ratio comparable to monoculture studies. (C) When calculating transmigration ratio and trajectory statistics, we find that the presence of BPLCs dampens PMN transmigratory behavior. For both sided EECM-BMEC-like cell stimulation conditions (10 pg/ml cytomix), transmigration levels, crawling speed, and persistence are found to be statistically similar. Notably, sided stimulation of EECM-BMEC-like cell resulted in similar PMN crawling speed and persistence when compared to results from negative control studies. In contrast, the presence of an fMLP gradient resulted in significantly faster PMN crawling speed (~22 μm/min). All results are plotted as mean ± SD and are analyzed with a 1-way ANOVA. Statistics: * = *P* ≤ 0.05, ** = *P* ≤ 0.01, *** = *P* ≤ 0.001, ns = not significant.

In conjunction with DS membrane materials, the incorporation of a BPLC culture limits the ability to analyze phase-contrast videos of PMN transmigration using automated methods [22] due to increased light scattering from the BPLCs, which affects video fidelity. Given this limitation, manual population counting and tracking were necessary (Fig. 7). Importantly, the presence of 3-μm pores enables the movement of PMNs across the silicon nanomembrane interface, where PMNs can then begin to interact with pericytes, emerge from the pericyte layer, and even “fall off” into the bottom channel (see Movie S8). Because transit through all these layers should naturally take longer than transit through one, we compared coculture and monoculture results by quantifying the loss of PMNs from the apical surface of the endothelium. In this way, any neutrophil diapedeses across the endothelium was classified “transmigrated” regardless of its exact sub-endothelial location (Fig. 7A).

Population counts were tabulated following the manual counting methodology described in prior research [22]. When all population counts in coculture experiments were normalized, averaged, and fit to a linear model, the average population showed a minimal decrease over time (Fig. 7B). PMN cell count within the recording FOV can be maintained for over 30 min as few cells migrate across the BPLC layer. This result facilitates our ability to compare transmigration on DS to studies on NPN membranes where the number of cells in the FOV does not change, owing to the impassibility of the nanopores in the NPN membrane.

Transmigration data shows that PMNs are again sensitive to fMLP in the coculture model, with an average transmigration ratio of 37.58% versus 4.10% in the negative control. The sided-cytomix (10 pg/ml) treatments were also statistically similar to one another, indicating that the direction of an inflammatory stimulation no longer affected PMN transmigration response when BLPCs were present (Fig. 7C). Additionally, PMN speed was equivalent across all tested conditions apart from fMLP-stimulated PMNs, which were faster than any other group (Fig. 7C). Interestingly, PMN persistence in coculture devices was statistically similar across all studies, indicating that the overall population motility was also similar between the apical and basal cytomix conditions.

### BPLCs dampen PMN transmigration response in a model of sepsis-like inflammation at the BBB

**Fig. 8.**
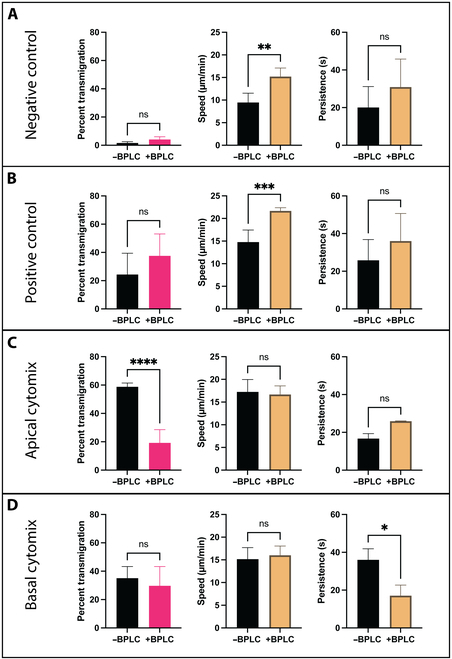
The addition of a BPLC culture elicits less PMN transmigration in response to sepsis-like stimulation. (A and B) EECM-BMEC-like cell and BPLC cocultures exhibited minimal differences in PMN trafficking behavior in both positive and negative controls, except for a small but statistically significant increase in crawling speed (by ~5 μm/min in cocultures) was observed. (C) In contrast, the addition of a BPLC culture resulted in significantly dampened PMN transmigratory response under sepsis-like, or apical, inflammatory stimulus (~19%) when compared to EECM-BMEC-like cell monoculture results under the same stimulatory condition (~58%). No significant changes to crawling speed or persistence were seen. (D) Under neuroinflammatory-like, or basal, cytomix stimulation, BPLC culture had no effect on PMN transmigratory behavior or crawling speed. The addition of BPLCs did result in a significant decrease in persistence, however. All results are plotted as mean ± SD and are analyzed with a 1-way ANOVA. Statistics: * = *P* ≤ 0.05, ** = *P* ≤ 0.01, *** = *P* ≤ 0.001, **** = *P* ≤ 0.0001, ns = not significant.

To better appreciate the impact of adding BPLCs to EECM-BMEC-like cell cultures on PMN transmigration, we directly compared results for monoculture (−BPLC) and coculture (+BPLC) in Fig. 8. This comparison makes clear how the negative control (nonstimulated) studies showed similar (and negligible) PMN transmigration levels and statistically similar persistence between both culture groups (Fig. 8A). Interestingly, PMNs in coculture devices were significantly faster (15.19 μm/min) than PMNs crawling in monoculture devices (9.47 μm/min) for the negative control studies (Fig. 8A). PMNs in μSiM-MVM devices with fMLP gradients incorporated into the bottom chamber displayed a similar trend to the negative control studies, where transmigration and persistence were statistically similar between the 2 culture groups and PMNs were again faster in coculture (21.67 μm/min) versus monoculture (14.78 μm/min) devices (Fig. 8B).

The most intriguing difference between monoculture and coculture conditions was that PMN transmigration was reduced by ~67% in coculture versus monoculture following apical cytomix (10 pg/ml) stimulation (Fig. 8C; no statistical differences between crawling speed and persistence; see also Fig. S7). In contrast, the addition of a BPLC culture had no significant effect on PMN transmigration when both monoculture and coculture devices were stimulated with basally applied cytomix (Fig. 8D), even though there was a significant decrease in persistence. Thus, the BPLCs appeared to provide a resilience to the barrier function under sepsis-like inflammatory conditions.

## Discussion

A tissue chip model of the BBB was developed, characterized, and validated for studies of barrier dysfunction during sepsis-like inflammation. We used hiPSCs differentiated into BMECs [19,23] and isogenically matched BPLCs [20,24]. This approach can eventually be used to study patient-specific BBB responses to inflammation and to probe at mechanistic contributions of each cell type under healthy and diseased conditions. While other models have evaluated immune cell migration in coculture [39,40], analyses are often limited to immune cell adhesion and migration. In animal studies, pericyte detachment in the brain during sepsis is correlated to increased leakage and immune cell migration [15], but findings from animal studies are often not borne out in human disease, and studies of human mechanisms in situ are limited because of the need to ensure patient safety. Using the μSiM and live cell imaging, we have evaluated immune cell migratory parameters in a human system and identified the contribution of pericytes to neutrophil migration under sepsis-like and neuroinflammation-like conditions.

Brain pericytes play an important role in the BBB and are necessary for normal physiological function. Importantly, pericyte depletion in disorders such as sepsis [11,15] is linked to the pathogenesis of numerous CNS sequelae [16]. Here, we compared EECM-BMEC-like cell monocultures and coculture with BPLCs against one another in permeability assays and leukocyte trafficking studies. While higher concentrations of cytomix have been used by others [31,39], the tolerated concentrations of the 3 cytokines in our model are within the ranges found in plasma of sepsis patients, although these levels are highly variable across patients and studies (varying from 1 to 25 pg/ml) [32,33]. Based on previous in vitro [41] and in vivo [15,42] studies, we initially hypothesized that addition of BPLCs would stabilize EECM-BMEC monolayers, possibly reducing baseline permeability and protecting against permeability increases in response to sepsis-like stimuli [20], but this was not what we observed. Our results are consistent with prior work using the same differentiation protocol for EECM-BMEC-like cells, which also found no impact on permeability with pericyte coculture [19]. These protocols were selected based on their ability to produce cells with brain-like characteristics that are suitable for immune cell-trafficking studies. Furthermore, the effect of pericytes on BBB permeability in vitro has had highly variable results depending on cell types and culture conditions. This may be due, in part, to high heterogeneity of pericytes in vivo and our inability to capture this in our hiPSC-based in vitro model. Future studies could compare results to primary pericytes and alternative brain pericyte differentiation protocols. Additionally, incorporation of other critical support cells, such as astrocytes, as well as physiological flow may improve the model.

Despite the lack of an effect of BPLCs on EECM-BMEC-like cell permeability responses, we found a pronounced contribution by BPLCs to basement membrane deposition. In the brain, the endothelial/pericyte basement membrane is composed primarily of collagen type IV, laminin, nidogen, perlecan, and fibronectin [43]. In these studies, we assessed the deposition of collagen type IV, laminin, and fibronectin. In agreement with animal studies, collagen type IV was produced by both endothelial cells and pericytes in our model [44,45]. Laminin is also produced by both cell types in the brain, although different isoforms are produced by each cell [43]. In the μSiM-BPB, a rich laminin layer was seen under the EECM-BMEC-like cell layer, but since we used a polyclonal antibody, we were not able to distinguish laminin isoforms. Future work should investigate this directly. We would expect the endothelial cell-deposited laminin layer to be composed primarily of laminin-411 and laminin-511 [43]. RNAseq data indicated that our BPLCs expressed less laminin subunit alpha-2 than primary human brain pericytes (Fig. S2), and this may explain the low levels of laminin in the BPLC layer of the μSiM-BPB. In rodent brains, fibronectin is primarily present during development and in disease states but less so in mature brains [43]. However, while the expression of fibronectin in our differentiated BPLCs is higher than the published data for BPLCs [20], it is not significantly different from primary brain pericytes (Fig. S2). Additionally, while basement membrane degradation is common in disease, and pericytes down-regulate components of the basement membrane upon exposure to cytokines [40], basement membrane degradation was not seen after a 16- to 20-h exposure to cytomix in our model. It is possible that longer stimulation is required to see basement membrane degradation by MMPs, or certain components are lacking in our model necessary to activate MMPs. Additional cell types may be necessary to reproduce this phenotype.

Our observations of different growth patterns using overgrown BPLC cultures on DS versus NPN membranes provides indirect evidence that physical cell–cell interactions occur between the 2 cell layers at the micropores in DS membranes. Future studies should evaluate the exact nature of these interactions. For example, Förster resonance energy transfer or proximity ligation assays could be used to compare expression of gap junction proteins or integrins across the micropores compared to the nanoporous regions of DS membranes. Functional studies could include LY microinjections, to evaluate whether the dye injected into endothelial cells enters pericytes. We could also block different integrins to see if adhesion patterns of the pericytes change. Another option takes advantage of the optical clarity of silicon nitride membranes, which are compatible with electron microscopy platforms. Techniques such as focused ion beam scanning electron microscopy have already been used to generate volumetric sections of brain tissue at high resolution (4 nm) and can be applied on the μSiM platform to check for endothelial cell–pericyte contact. While evaluation of these potential contact points was beyond the scope of the current work, future studies should explore the form and nature of these contacts and may set apart the DS nanomembrane materials from alternate porous substrates.

PMN trafficking was evaluated to model immune cell infiltration across the BBB during sepsis-like and neuroinflammation-like stimulation. In monoculture studies, we observed significantly higher PMN trafficking under sepsis-like inflammation versus tissue-sided stimulation for both low (1 pg/ml) and high (10 pg/ml) cytomix concentrations. This behavior was surprising and is opposite of what has been observed in similar experiments that utilized a human umbilical vein endothelial cell (HUVEC) monolayer in PMN trafficking analysis [17], which may be attributed to the use of TNF-α versus cytomix. Differences in endothelial cells from different tissues or vessel types are well documented, and different cytomix responses for cells with different tissue origins have also been published [46]. Thus, it is possible that intrinsic mechanisms may make EECM-BMEC-like monolayers more susceptible to peripheral inflammatory factors than HUVECs.

One limiting factor that emerged in our study was the inability to use our ML protocol [22] to analyze PMN trafficking videos on BPLC cocultures, necessitating a tedious manual analysis. This is due to the introduction of new image elements (e.g., pericytes and micropores) that dramatically changed the morphological feature profile of the recorded videos. Ultimately, this served to make EECM-BMEC-like cells and neutrophils more difficult to detect and classify with our label-free imaging methods. Resolving this will require additional ground truth labeling of images to create masks with multiple classes. These, alongside the usage of ensemble models, would serve to retrain the ML algorithm for better accuracy. Given the time investment required to train and validate such a model, we chose to engage in manual analysis instead for this research. Future efforts will require an updated ML process to ensure rapid translation of video data into results.

The PMN tracking parameters extracted from monoculture and coculture studies were used to assess different PMN behaviors. Speed represents PMN crawling rates, while persistence is a value that describes a capability to maintain a direction of motion [25,27,28]. When analyzing these variables, a few notable patterns emerge. For both negative and positive control studies, PMNs crawled faster in coculture versus monoculture devices. In addition, PMN persistence was found to be statistically similar for both culture groups, indicating that the addition of BPLCs only increased baseline PMN crawling speed in control studies. In contrast, PMNs were shown to have similar (~15 μm/min) speed for all cytomix experiments, suggesting that the presence of a BPLC layer or directional cytokine stimulation is irrelevant for dictating PMN crawling speed when compared to monoculture studies alone. Interestingly, baseline PMN speed in the negative control group were also increased to ~15 μm/min with the addition of BPLCs. While the mechanism responsible for this crawling speed increase is unclear, pericytes are known to heavily interact with bloodborne leukocytes through secreted factors, including PMNs, and modulate their migratory behavior [11]. Thus, the inclusion of a BPLC culture itself may be enough to initiate the increased PMN crawling speed seen in this study. One interesting observation was seen in positive control coculture studies with fMLP, where PMNs were significantly faster (~20 μm/min) than all other tested conditions. Given that some subtypes of pericytes (e.g., NG2^+^) are known to be sensitive to fMLP and will secrete factors in response to it (e.g., IL-8) to attract PMNs [47,48], the resultant increase in PMN speed may be attributed to this interaction. For the cytomix studies, basal-sided stimulation resulted in a significant decrease in PMN persistence when a BPLC culture was added. Knowing that persistence correlates to the amount of time PMNs maintain a direction of motion, under a basal cytomix stimulation regime, BPLCs likely direct PMNs across the endothelial barrier in a more efficient manner. While the role of pericytes in directing and modifying leukocyte trafficking is well documented [11,40,48], it is unclear how endothelial cell apicobasal polarity and sided inflammatory stimulus modulate this behavior. Future studies will need to probe the underlying molecular mechanisms behind such interactions.

A key finding from the neutrophil transmigration experiments is that addition of BPLCs resulted in a significantly reduced rate of PMN transmigration under sepsis-like conditions compared to EECM-BMEC-like monolayers on their own. This is in line with previous findings in the literature that evaluated leukocyte trafficking in Transwell-style models under similar inflammatory conditions [40]. Our results also coincide with observations made with in vivo studies where lipopolysaccharide was administered systemically in mice and loss of pericyte coverage was correlated with increased immune cell infiltration [15]. Conversely, we found that the addition of BPLCs had no effect on leukocyte trafficking numbers when the inflammatory stimulus originated from the basal compartment, even though there was a reduction in PMN persistence. These findings were made possible by use of the μSiM platform and its highly permeable nanomembranes and glass-like imaging properties. Our results suggest that pericytes may play a role in protecting the CNS from excessive leukocyte infiltration during sepsis-like inflammation but not during neuroinflammation. Future study is still required to understand the mechanisms underlying these differential behaviors, which may be related to pericyte secretion of additional cytokines and chemokines, cell–cell communication impacting endothelial expression of adhesion molecules, or an impact of the pericyte contribution to the basement membrane. It will also be critical to assess whether the incorporation of additional peripheral cells (e.g., astrocytes and glial cells) and mechanical stresses such as fluid flow into the model will further modulate the response to inflammatory stimuli. With these future studies, it may be possible to identify therapeutic targets to prevent excessive immune cell migration into uninfected tissues during sepsis, without impacting the ability of immune cells to locate and resolve the causative infection.

## Data Availability

The datasets used and/or analyzed during the current study are available from the corresponding author on reasonable request.
