# Supplementary material for: Pericytes Enrich the Basement Membrane and Reduce Neutrophil Transmigration in an In Vitro Model of Peripheral Inflammation at the Blood–Brain Barrier
Source: Biomater Res. 2024 Oct 3;28:0081. doi: 10.34133/bmr.0081 (PMC11447289; doi:10.34133/bmr.0081)
Supplement: Supplementary 1 — Table S1 Figs. S1 to S7 Movie S1 [file bmr.0081.f1.zip › CocultureSepsis_Manuscript_Supplement_R4.docx]

**Front Matter**

SUPPLEMENTARY MATERIALS

Title

Full: Pericytes enrich the basement membrane and reduce neutrophil transmigration in an in vitro model of peripheral inflammation at the blood brain barrier

Short: Pericytes shield brain in inflammatory model

**Authors**

Molly C. McCloskey^1†^, S. Danial Ahmad^1†^, Louis P. Widom^2^, Pelin Kasap^3^, Benjamin D. Gastfriend^4,5^, Eric V. Shusta^4,6^, Sean P. Palecek^4^, Britta Engelhardt^3^, Thomas R. Gaborski^2^, Jonathan Flax^1^, Richard E. Waugh^1^, James L. McGrath^1*^

**Affiliations**

† = These authors contributed equally to this work

* = Address correspondence to: jmcgrath@ur.rochester.edu

^1^ = Department of Biomedical Engineering, University of Rochester, Rochester NY, USA.

^2^ = Department of Biomedical Engineering, Rochester Institute of Technology, Rochester NY, USA.

^3^ = Theodor Kocher Institute, University of Bern, Bern, Switzerland.

^4^ = Department of Chemical and Biological Engineering, University of Wisconsin–Madison, Madison, WI, USA.

^5^ = Departments of Pharmacology and Neurosciences, University of California, San Diego, La Jolla, CA, USA.

^6^ = Department of Neurological Surgery, University of Wisconsin–Madison, Madison, WI, USA.

**Table S1.** Antibodies used for immunofluorescence staining

| **Antibodies** | **Fixative** | **Clone** | **Source** | **Cat. N.** | **Dilution** |
| --- | --- | --- | --- | --- | --- |
| Mouse Anti-Human ICAM-1 IgG1, κ | live | HA58 | BioLegend | 353102 | 1:100 |
| Mouse Anti-Human Claudin-5 IgG1 | MeOH | 4C3C2 | Invitrogen | 35-2500 | 1:200 |
| Rabbit Anti-Human PDGFRβ IgG | MeOH | 28E1 | Cell Signaling Technology | 3169 | 1:100 |
| Mouse Anti-Human Collagen Type IV IgG2b, κ, Alexa Fluor 647 | live | 1042 | Invitrogen | 51-9871-82 | 1:100 |
| Rabbit Anti-Human Fibronectin IgG1, Alexa Fluor 488 | live | FN-3 | Invitrogen | 53-9869-82 | 1:200 |
| Rabbit Anti-Human Laminin IgG | live | Polyclonal | Invitrogen | PA1-16730 | 1:100 |
| Goat Anti-Mouse IgG Alexa Fluor 488 | N/A | N/A | Invitrogen | A11001 | 1:200 |
| Goat Anti-Rabbit IgG Alexa Fluor 568 | N/A | N/A | Invitrogen | A11011 | 1:200 |

**
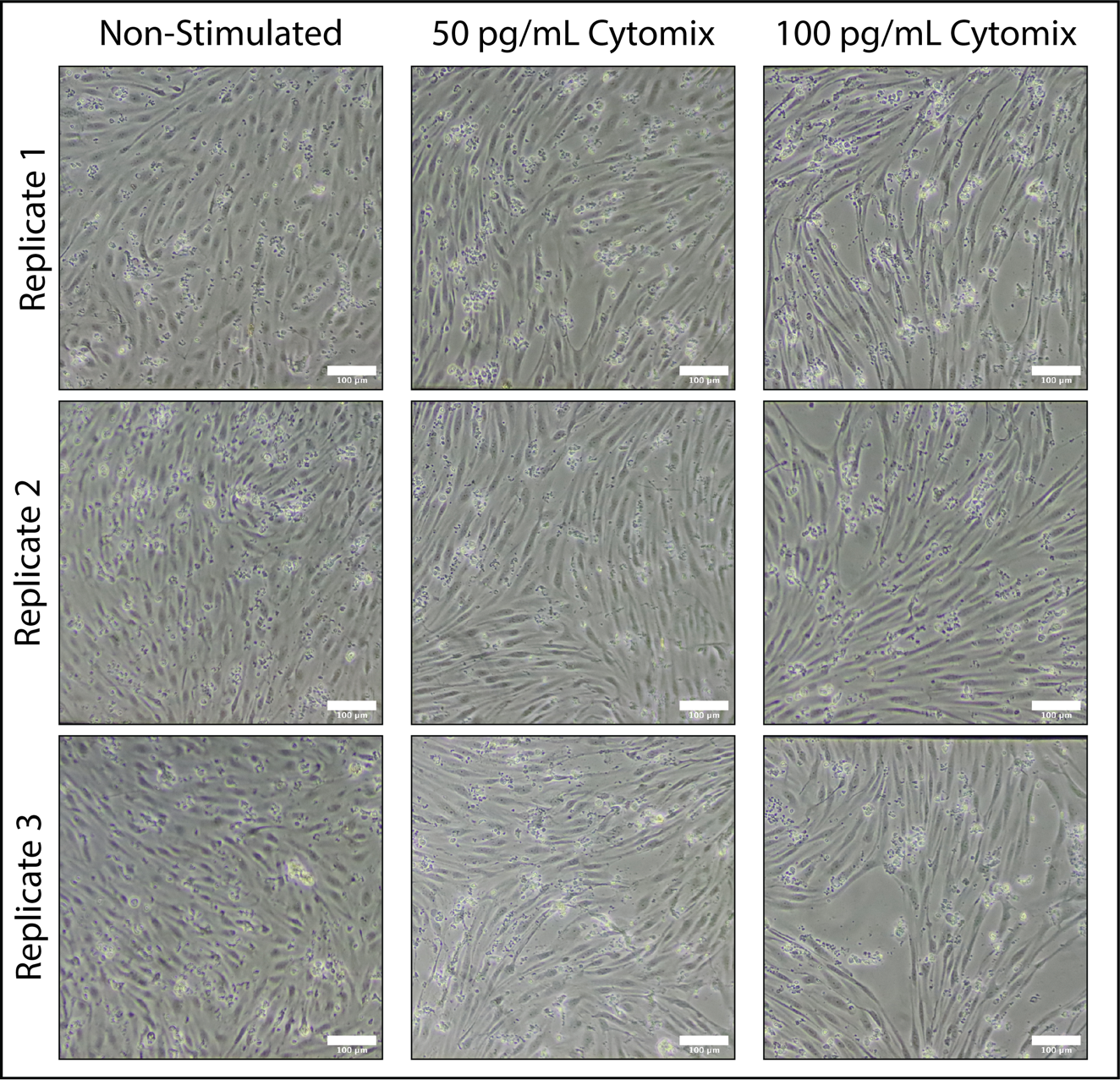
**

**Fig. S1** Cytomix optimization. Phase images of EECM-BMEC-like cells cultured in µSiMs for 6 days and treated for 16-20 hour with cytomix (equimolar TNF-⍺ + IFN-𝛾 + IL-1β at 50 pg/mL or 100 pg/mL each cytokine) or media (Non-Stimulated). Both concentrations of cytomix resulted in several large gaps for multiple devices and in some cases appeared to cause cell death, indicating lower stimulant concentrations were necessary. Scale bar = 100 µm.

**
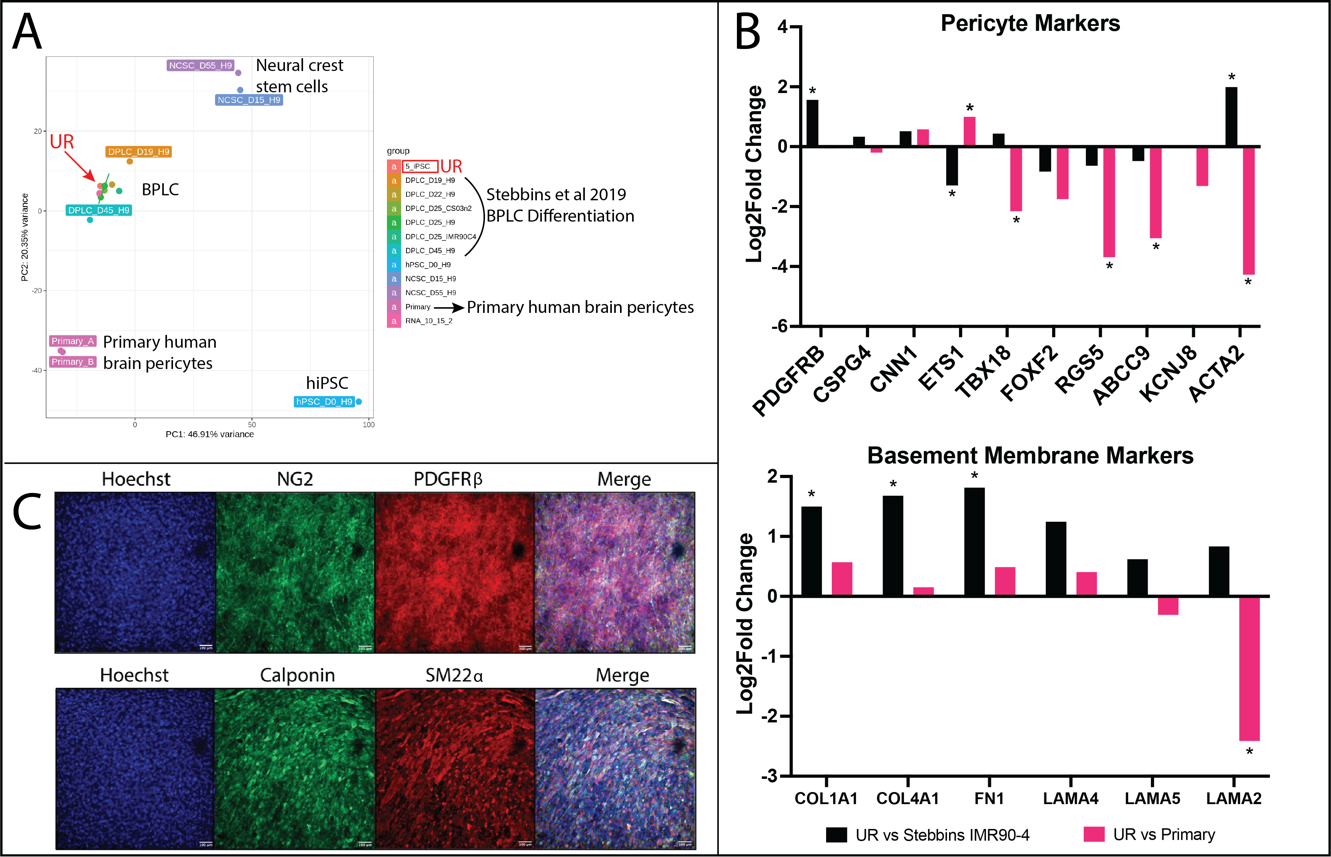
**

**Fig. S2** Molecular characterization of brain pericyte-like cells (BPLC). (**A**) PCA plot comparing RNAseq data from BPLCs differentiated at the University of Rochester (UR) to data published in Stebbins et al., 2019^1^, which includes BPLCs generated from multiple clones at different stages of differentiation, neural crest stem cells (NCSC), undifferentiated human induced pluripotent stem cells (hiPSC), and primary human brain pericytes. The BPLCs used in these studies are marked ‘UR” and highlighted in red. (**B**) RNAseq was performed and expression of pericyte markers and basement membrane markers for the BPLCs generated at UR were compared to data from the original publication, namely BPLCs derived from IMR90-4 (black) and primary human brain pericytes (pink). Log2FoldChange is plotted, with asterisks indicating significantly different expression, adjusted P ≤ 0.05. (**C**) Representative images of BPLCs cultured in tissue culture plates and stained for pericyte markers, NG2, PDGFRβ, Calponin, and SM22⍺, and nuclear stain Hoechst. Scale bar = 100 µm.

**
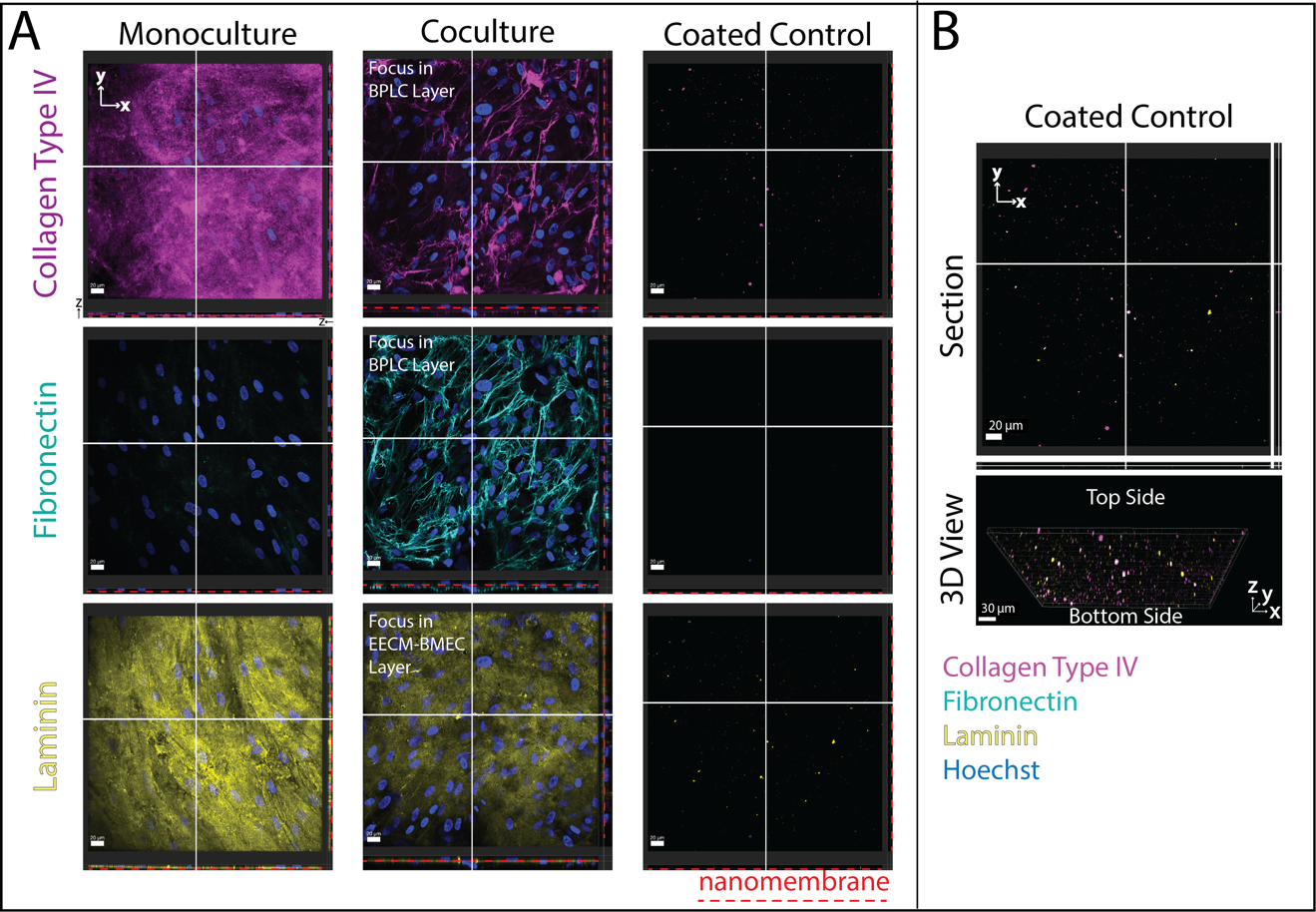
**

**Fig. S3** Confocal imaging of individual basement membrane proteins deposited in the µSiM-BPB and on coated control µSiMs. EECM-BMEC-like cells (Monoculture) and EECM-BMEC-like cells and BPLCs (Coculture) were grown in µSiMs and stained for basement membrane components collagen type IV (magenta), fibronectin (teal) and laminin (yellow), and nuclear stain Hoechst (blue), along with a collagen type IV and fibronectin coated control device. Confocal microscopy was used to acquire images across the nanoporous membrane region. (**A**) Representative monoculture, coculture, and coated control Section confocal images, which show the xy-plane (middle), the xz-plane (below), and yz-plane (right). The white lines show the location of the xz and yz sections. The approximate location of the nanomembrane across the z axis is indicated by a red dashed line. Scale bar = 20 µm. (**B**) Representative Section and 3D View images of a coated control device. The Section image (scale bar = 20 µm) shows the xy-plane (middle), the xz-plane (below), and yz-plane (right). The white lines show the location of the xz and yz sections. The 3D View (scale bar = 30 µm) shows the entire confocal stack, focused below the nanomembrane, with the “Bottom Side” indicating the channel, and the “Top Side” indicating the top well.

**
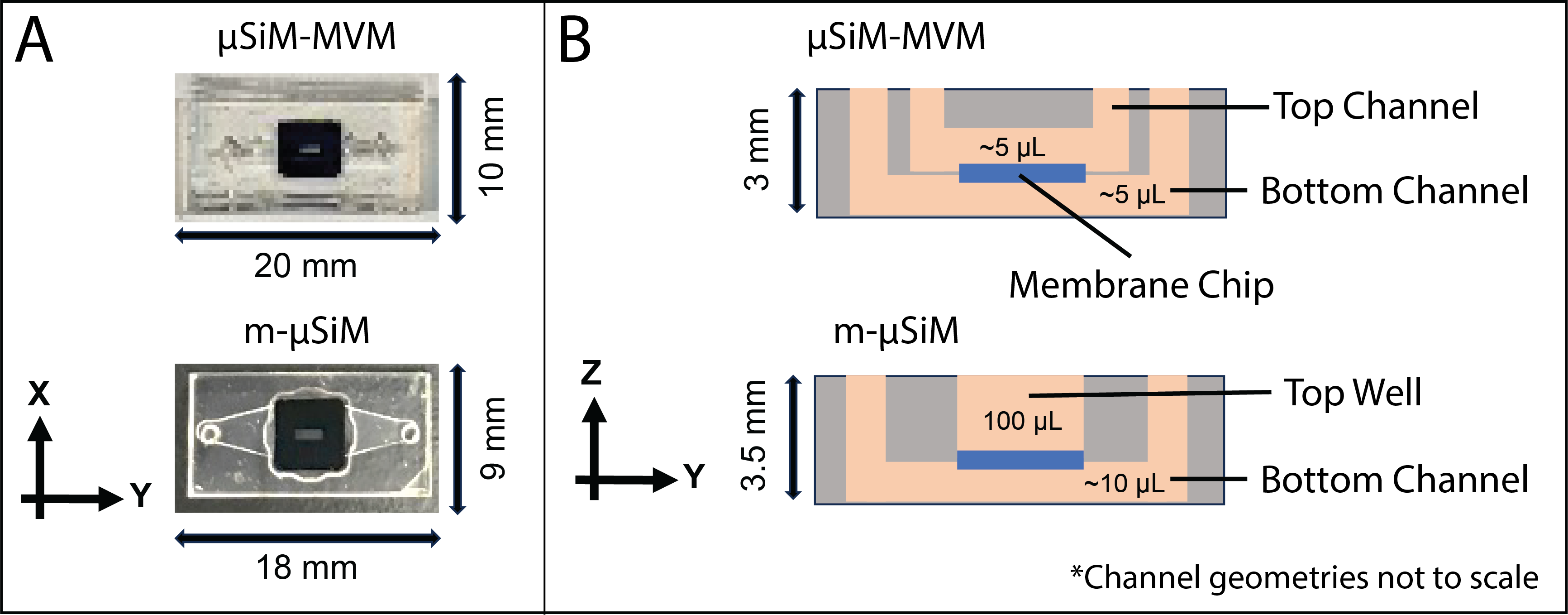
**

**Fig. S4** µSiM-microvascular mimetic (µSiM-MVM) versus modular µSiM (m-µSiM) designs. (**A**) Images of the µSiM-MVM and m-µSiM taken from the top surface, or xy-plane. The overall dimensions of the devices are similar. (**B**) Sideview, or yz-plane, schematics of the µSiM-MVM and m-µSiM. The volumes of the µSiM-MVM are notably smaller than the m-µSiM, but the closed channel geometry enables high quality live imaging for immune cell transmigration studies.

**
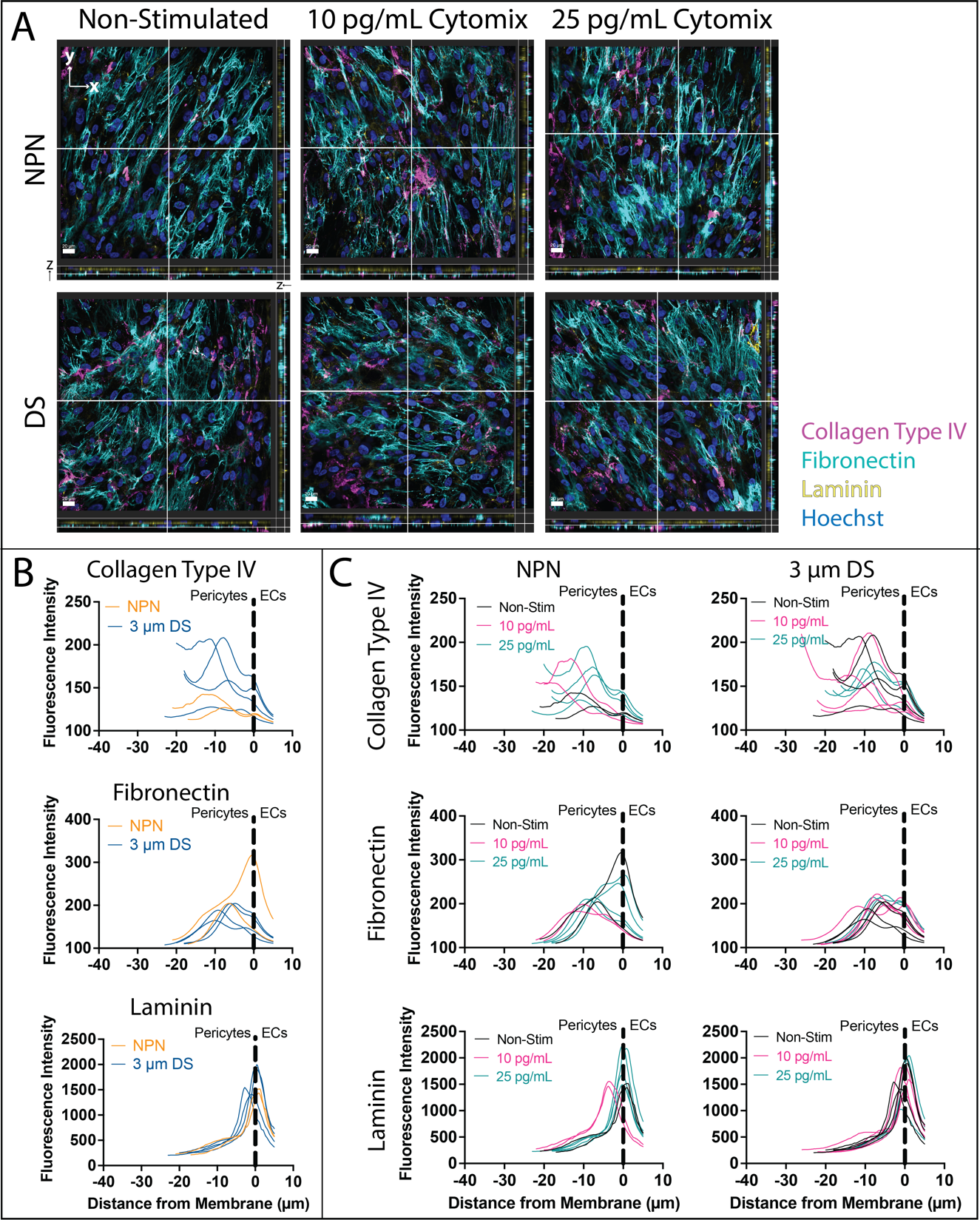
**

**Fig. S5** Characterization of the µSiM-BPB on NPN and 3 µm dual-scale (DS) nanomembranes with and without cytomix stimulation. EECM-BMEC-like cells were cocultured with BPLCs on nanoporous (NPN) or dual-scale (DS) membranes and either not stimulated (Non-Stimulated) or treated for 16-20 hour with cytomix at 10 pg/mL or 25 pg/mL. Following a permeability assay with lucifer yellow, devices were stained for basement membrane components collagen type IV (magenta), fibronectin (teal) and laminin (yellow), along with nuclear stain Hoechst (blue). Confocal microscopy was used to acquire images across the nanoporous membrane region. Remaining lucifer yellow from the permeability experiment may have impacted fibronectin staining and quantification since the fluorophore conjugated to the anti-fibronectin antibody had similar excitation/emission wavelengths to the lucifer yellow. (**A**) Representative section images are shown, displaying the xy-plane (large), the xz-plane (below), and yz-plane (right). The white lines show the location of the xz and yz sections. All images are focused within the bottom chamber pericyte layer along the z axis. Scale bar = 20 µm. (**B**) EECM-BMEC-like cells were cocultured with BPLCs on nanoporous (NPN, orange) or 3 µm dual-scale (DS, blue) membranes. Mean fluorescence intensity across the z axis was measured and plotted for each basement membrane protein. (**C**) EECM-BMEC-like cells were cocultured with BPLCs on nanoporous (NPN) or dual-scale (3 µm DS) membranes and either not stimulated (Non-Stim, black) or treated for 16-20 hour with cytomix at 10 pg/mL (pink) or 25 pg/mL (teal). Mean fluorescence intensity across the z axis was measured and plotted for each basement membrane protein. N = 1-2 devices with 1-2 images acquired per device.

**
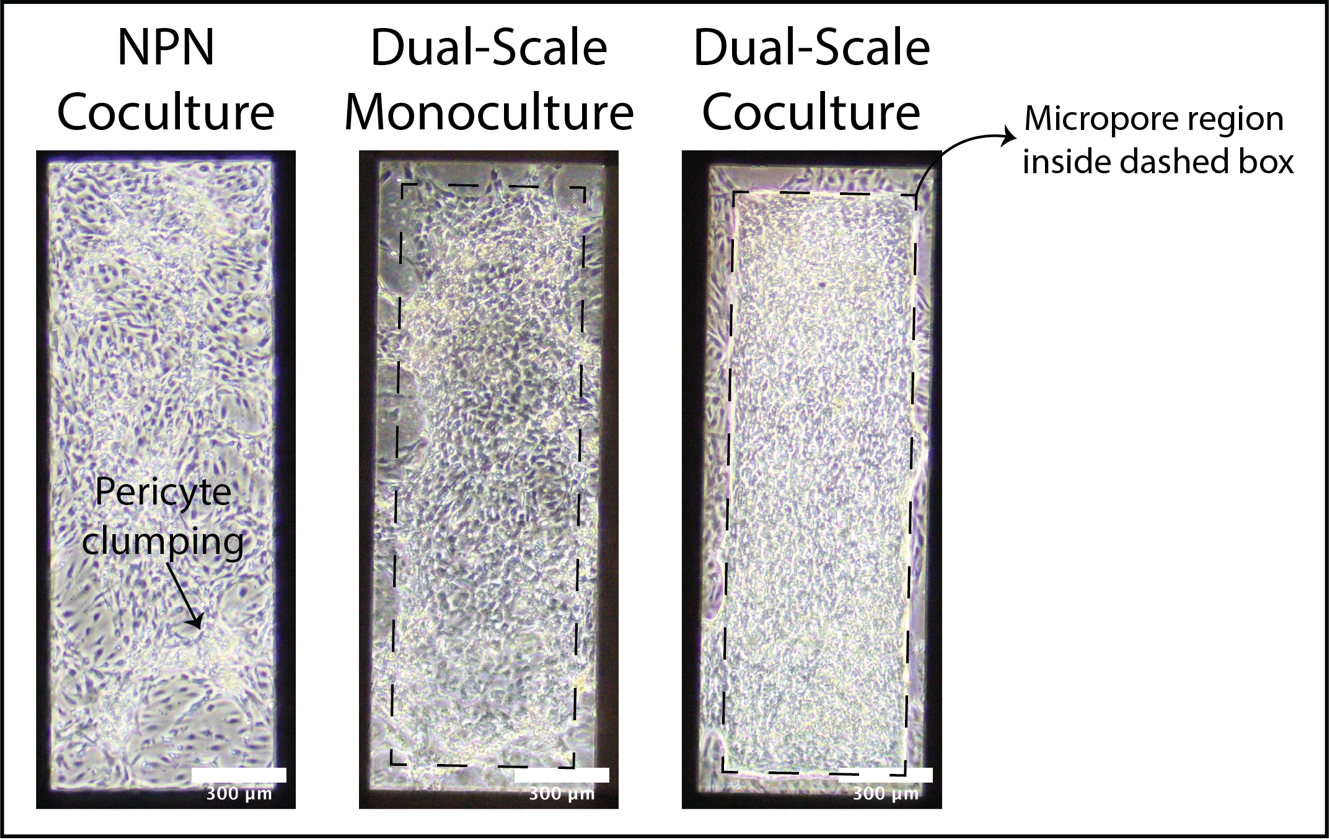
**

**Fig. S6** Evidence for cell-cell contact on coculture dual-scale membranes. EECM-BMEC-like cells and BPLCs (Coculture) were grown in µSiMs on nanoporous (NPN) and 3 µm dual-scale membranes, and BPLCs alone (Monoculture) were cultured on dual-scale membranes. Dual-scale membranes contain 3 µm pores etched onto a nanoporous background but are offset from the membrane edge to improve stability. The region containing micropores is outlined in a black dashed line. BPLCs were overseeded and grown several days past confluency. Pericytes grown across NPN membranes, which do not have micropores to enable cell-cell contact, peel off from the membrane and begin to clump in the middle. Similarly, pericytes grown across dual-scale membranes without endothelial cells to adhere to on the opposite chamber pull back beyond the start of the micropore region of the membrane. Pericytes grown across dual-scale membranes, which do have micropores to enable cell-cell contact with endothelial cells in the opposite chamber, adhere to the micropore region of the nanomembrane in coculture. This is evidence, but not definitive, that cell-cell contact occurs across dual-scale, but not nanoporous membranes. Scale bar = 300 µm.

**Fig. S7** The presence of BPLCs results in a reduction in PMN migration under apical stimulation. To support the conclusion that the presence of BPLCs affects PMN transmigration differently under apical versus basal cytomix stimulation, a multifactorial strategy was used to assess all experimental groups together. This was done to avoid errors attributed to an approach called “differences in nominal significance” or DINS^2^, where only one factor (stimulation sidedness or BPLC presence) were compared. All results are plotted as mean ± SD and are analyzed with a two-way ANOVA with interaction term. Statistics: * = P ≤ 0.05, ** = P ≤ 0.01, *** = P ≤ 0.001, **** = P ≤ 0.0001, ns = not significant.

**Video S1** PMNs were incorporated into a µSiM-BPB featuring an apically stimulated (10 pg/mL) BMEC-BPLC coculture. Despite the presence of pericytes, PMNs are capable of transmigrating fully across the pericyte layer, as indicated by the defocus ring artifacts seen when PMNs fall into the bottom of the device.

References

1. Stebbins MJ, Gastfriend BD, Canfield SG, Lee MS, Richards D, Faubion MG, Li WJ, Daneman R, Palecek SP, Shusta EV. Human pluripotent stem cell-derived brain pericyte-like cells induce blood-brain barrier properties. Sci Adv 2019;5(3):eaau7375.

2. Vorland CJ. Sex difference analyses under scrutiny. Elife 2021;10.
